# Risk of early neurodevelopmental disorders associated with in utero exposure to valproate and other antiepileptic drugs: a nationwide cohort study in France

**DOI:** 10.1038/s41598-020-74409-x

**Published:** 2020-10-22

**Authors:** Joël Coste, Pierre-Olivier Blotiere, Sara Miranda, Yann Mikaeloff, Hugo Peyre, Franck Ramus, Mahmoud Zureik, Alain Weill, Rosemary Dray-Spira

**Affiliations:** 1 Department of Public Health Studies, French National Health Insurance (CNAM), Paris, France; 2 Université de Lorraine, Université Paris-Descartes, Apemac, 4360 Nancy, EA France; 3 Department of Epidemiology of Health Products, The French National Agency for Medicines and Health Products Safety (ANSM), Saint-Denis, France; 4 Hôpital Bicêtre, INSERM, Université Paris-Saclay, Villejuif, France; 5 Department of Child and Adolescent Psychiatry, Hôpital Robert Debré, Paris, France; 6 Laboratoire de Sciences Cognitives Et Psycholinguistique, Ecole Normale Supérieure, EHESS, CNRS, PSL University, Paris, France; 7 INSERM UMR 1141, Paris Diderot University, Paris, France; 8 Versailles Saint-Quentin University, Montigny le Bretonneux, France

**Keywords:** Epidemiology, Neurodevelopmental disorders, Adverse effects

## Abstract

Information available on the risks of neurodevelopmental disorders (NDs) associated with in utero exposure to valproate (VPA) and to other antiepileptic drugs (AEDs) is limited. A nationwide population-based cohort study was conducted based on comprehensive data of the French National Health Data System (SNDS). Liveborn infants without brain malformation, born between January 2011 and December 2014, were followed from birth up to December 2016. NDs were identified based on diagnoses of mental or behavioural disorders and utilization of speech therapy, orthoptic or psychiatric services. The risk of NDs was compared between children exposed in utero to AED monotherapy and unexposed children, using Cox proportional hazard models adjusted for maternal and neonatal characteristics. The cohort included 1,721,990 children, 8848 of whom were exposed in utero to AED monotherapy. During a mean follow-up of 3.6 years, 15,458 children had a diagnosis of mental or behavioural disorder. In utero exposure to VPA was associated with an increased risk of NDs overall (aHR: 3.7; 95% CI 2.8–4.9) and among children born to a mother without mental illness (aHR 5.1; 95% CI 3.6–7.3). A dose–response relationship was demonstrated and the risk of NDs was more particularly increased for an exposure to VPA during the second or third trimesters of pregnancy. Among the other AEDs, only pregabalin was consistently associated with an increased risk of NDs (aHR: 1.5; 95% CI 1.0–2.1). This study confirms a four to fivefold increased risk of early NDs associated with exposure to VPA during pregnancy. The risk associated with other AEDs appears much lower.

## Introduction

Epilepsy is one of the most common conditions affecting women of reproductive age^1,2^, and most of them need antiepileptic drugs (AED) to avoid the harmful effects of uncontrolled seizures on themselves and their offspring^3^. However, in utero AED exposure is associated with congenital malformations of varying degrees of severity^4,5^ and with neurodevelopmental disorders (NDs). In utero exposure to valproate (VPA) is associated with poorer educational attainment^6–8^, poorer cognitive skills (IQ^9,10^ and language^10–13^, motor^13,14^, attentional^13,15,16^, behavioural^12,14,16–18^ and social skills^14–17^) and various NDs (mental retardation^18^, language disorders^12^, motor disorders^13^, attention-deficit/hyperactivity disorder^15^ and autism spectrum disorders^19^). These disorders may affect 30 to 40% of exposed children^16^, and their frequency increases with the dose of VPA administered to the mother^20,21^. However, previous studies have failed to determine whether the risk differs according to the period of exposure during pregnancy. Data concerning other AEDs are heterogeneous and insufficient to allow any definitive conclusions concerning the risk of NDs^18,20,21^. Although a number of studies have been conducted on this topic, it is difficult to draw firm conclusions given the variety of methods and outcomes considered, related to cognitive skills (e.g. IQ or language skills), NDs (e.g. autism spectrum disorders, or attention-deficit/hyperactivity disorder) or school performance.

The aim of the present study was to assess the risk of early NDs associated with in utero exposure: (1) to VPA used in epilepsy, in particular according to dose and period of exposure during pregnancy; and (2) to other AEDs.

## Methods

### Data sources

This study was conducted using the French national health data system (SNDS), including health insurance claim and hospital discharge databases linked by a unique patient identifier. The system covers the entire French population and contains comprehensive data on all reimbursements for health-related expenditures^22^, including dispensed drugs (coded using the Anatomical Therapeutic Chemical, ATC, classification) with date of dispensing and outpatient care performed by healthcare professionals, as well as demographic data such as sex, age, area of residence, vital status, and Complementary Universal Health Insurance scheme (CMU-C, a system providing free access to healthcare for people with an annual income below 50% of the poverty threshold). Patients’ status for 100% reimbursement of care related to a severe and costly long-term disease (LTD) is recorded and coded according to the International Statistical Classification of Diseases and Related Health Problems, Tenth Revision (ICD-10). The hospital discharge database provides detailed information about all admissions to French public and private hospitals since 2006. Diagnoses (ICD-10 coded) and medical or surgical procedures performed during hospital stays are available. Mother–child data linkage, possible since 2011 for deliveries in a public institution and since 2012 for all deliveries, was available for 94% of all deliveries in 2014.

This study was approved by the French Data Protection Supervisory Authority (*Commission Nationale de l’Informatique et des Libertés*). No informed consent is required for studies based on French administrative medical databases, as these data are anonymous.

### Study population and follow-up

All liveborn singleton children born between 1st January 2011 and 31st December 2014 were eligible for inclusion. The mother had to be covered by the national health insurance general scheme for salaried workers (which represents 75% of the French population) and to have had at least one health expenditure reimbursement over the 2 years preceding the onset of pregnancy. We excluded children who could not be linked to their mother’s data, those with missing data for sex, gestational age and birth weight and those with a diagnosis of brain malformation (ICD-10 codes Q00 to Q04 and Q05.0 to Q05.4) during their stay in the maternity unit. When the mother had several deliveries during the study period, only the first single liveborn child during the study period was included.

Children were followed from birth until 31/12/2016 (study end date) or until the first of the following conditions, when it occurred before the end of the study: (i) occurrence of the event of interest; (ii) child lost to follow-up, defined as a period of more than 12 months without any reimbursement; (iii) child’s death.

### Exposures

Among VPA products, only those indicated for the treatment of epilepsy, i.e. those containing valproic acid and sodium valproate, were considered. The other AEDs considered were lamotrigine and carbamazepine (also indicated in bipolar disorder), clonazepam, pregabalin and gabapentin (also indicated in neuropathic pain), levetiracetam, oxcarbazepine, phenobarbital, and topiramate (also indicated in migraine). In utero exposure to each AED was defined by at least one dispensing of the drug to the mother between the beginning of the month preceding onset of pregnancy and the end of pregnancy. Children considered to be exposed to each AED were those whose mother had used this drug as monotherapy, defined by the use of a single drug indicated for epilepsy during pregnancy (children whose mother had used several AEDs during pregnancy were excluded due to the major difficulty of measuring the respective association of each drug with the risk of NDs). The period of exposure to the AED during pregnancy was categorized as follows: exposure exclusively during the 1st trimester, exposure during the 1st trimester and during the 2nd or 3rd trimester, exposure exclusively during the 2nd or 3rd trimester. The mean daily dose and the cumulative dose of each AED during pregnancy were estimated from the total quantity of drug substance dispensed and the number of days covered during pregnancy, and categorized according to the tertiles of their distributions.

### Outcomes

NDs were assessed based on diagnoses (and age at first diagnosis) of mental and behavioural disorders, identified by the attribution of LTD status and/or hospital admission (ICD-10 code F70 to F98). The following diagnostic categories were distinguished: “pervasive developmental disorders” (F84, corresponding to autism spectrum disorders in ICD-11 [6A02]), “mental retardation” (F70–F79), “disorders of psychological development” (F80–F89, including language, motor, learning and autism spectrum disorders), and “behavioural and emotional disorders with onset usually occurring in childhood and adolescence” (F90–F98). Despite the imperfect match with ICD-11 categories, for convenience we collectively refer to categories F70–F98 as “neurodevelopmental disorders” in this paper. We also assessed health care utilization (and age of first utilization) in office medicine or in the outpatient department of a public hospital, identified by reimbursement of at least (i) one speech therapy session; (ii) one orthoptist consultation; (iii) one psychiatrist or child psychiatrist consultation.

### Covariates

Information was available on the following characteristics known or likely to be related to both AED therapy in the mother and NDs in the offspring: mother’s age and CMU-C status at the time of delivery, mean monthly salary over the 3 months preceding maternity leave, year of the end of pregnancy, folic acid supplementation (folic acid dispensing during the month preceding pregnancy or the first trimester of pregnancy), alcohol use during the year preceding pregnancy or during pregnancy (in the mother: diagnosis of alcohol-related disease and/or dispensing of a drug indicated for the treatment of alcohol abuse; or in the neonate: diagnosis of maternal use of alcohol or foetal alcohol syndrome), tobacco use during the year preceding pregnancy or during pregnancy (in the mother: diagnosis of tobacco-related disease and/or dispensing of nicotine replacement therapy, a drug indicated for the treatment of tobacco dependence or a bronchodilator exclusively indicated in the treatment of chronic obstructive pulmonary disease; or in the neonate: diagnosis of maternal tobacco use), diagnosis of mental illness other than tobacco or alcohol use disorders, dispensing of psychotropic drugs (antidepressants, especially selective serotonin reuptake inhibitors [SSRI], antipsychotics, anxiolytics, hypnotics) during the year preceding pregnancy or during pregnancy, number of ATC classes of psychotropic drugs dispensed during the year preceding pregnancy (used as an indicator of severity of psychiatric morbidity); and child gestational age, sex, birth weight and neonatal diseases (diagnosis of cerebrovascular disease, intra-uterine hypoxia, birth asphyxia, intracranial haemorrhage).

### Statistical analysis

In the main analysis, incidence rate of the various outcomes was estimated for each exposure group and the risk was compared between children exposed in utero to monotherapy with each AED considered and unexposed children. The comparison group for VPA and AEDs not indicated in bipolar disorder (clonazepam, gabapentin, levetiracetam, oxcarbazepine, phenobarbital, pregabalin and topiramate) was composed of children not exposed to any AED during pregnancy. The comparison group for lamotrigine and carbamazepine was composed of children not exposed to either AED or any drugs for bipolar disorder during pregnancy. All these comparisons were performed globally for the overall study population, and by restricting the population to children born to a mother with no known mental illness, i.e. with no history of diagnosed mental illness and no dispensing of psychotropic drugs during the year preceding pregnancy.

In complementary analyses, the risk associated with each event studied was compared between: children exposed to VPA monotherapy and children exposed to lamotrigine monotherapy (i.e. with no other associated drug indicated for the treatment of epilepsy); between children exposed to VPA monotherapy categorized according to the dose of VPA during pregnancy and unexposed children; between children exposed to VPA monotherapy categorized according to the trimester of exposure to VPA during pregnancy and unexposed children.

Cox proportional hazard regression models were used to estimate adjusted hazard ratios (aHRs) and their 95% confidence intervals (CI) comparing the risk of the events studied according to AED exposure. All models were adjusted for the mother’s sociodemographic and medical characteristics (age, CMU-C status, folic acid supplementation, alcohol use, tobacco use, history of mental illness other than tobacco or alcohol use disorders, severity of psychiatric morbidity, psychotropic drugs during the year preceding pregnancy, selective serotonin reuptake inhibitor during pregnancy) and neonatal characteristics (sex, gestational age, birth weight).

### Ethics approval and consent to participate

This study was approved by the *Commission Nationale de l'Informatique et des Libertés* (French data protection agency) (regulatory decision DE-2011–078).

### Ethical publication statement

All authors confirm that they have read the Journal’s position on issues involved in ethical publication and affirm that this report is consistent with those guidelines.

## Results

**Figure 1:**
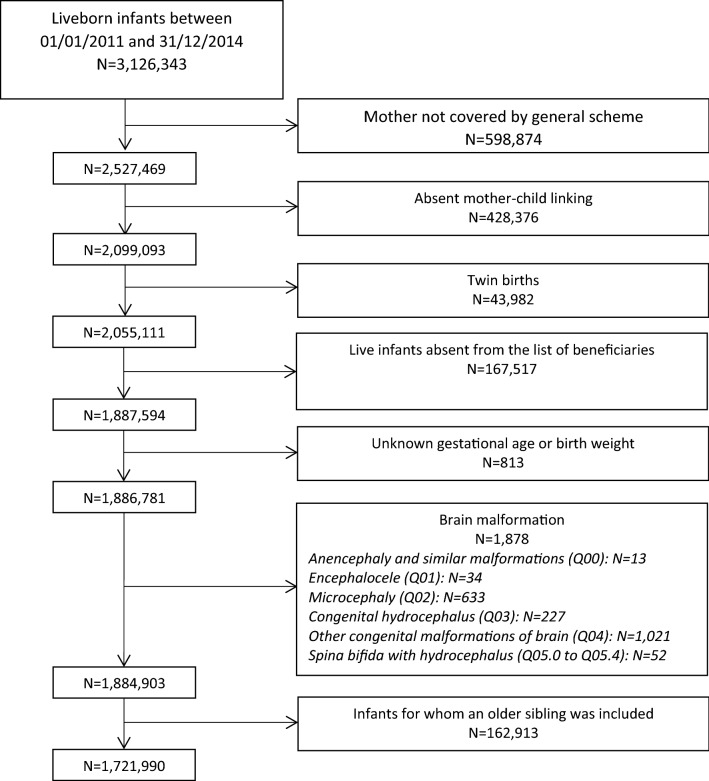
Flowchart of the study population.

The study population was composed of 1,721,990 single liveborn children between 01/01/2011 and 31/12/2014 (Fig. 1). Among the mothers who had used an AED during pregnancy (N = 11,549), 3862 had used lamotrigine, 1491 VPA, 1,777 pregabalin, 1559 clonazepam, 1210 levetiracetam, 791 carbamazepine, 697 topiramate, 467 gabapentin, 250 oxcarbazepine, and 154 phenobarbital. AEDs were predominantly used as monotherapy during pregnancy (Supplementary Table 1).

The majority (60.6%) of children exposed to VPA, lamotrigine, carbamazepine, levetiracetam, oxcarbazepine and phenobarbital during pregnancy were exposed for prolonged periods, i.e. during the 1st trimester and at least the 2nd or 3rd trimester of pregnancy. In contrast, in children exposed to clonazepam, gabapentin, pregabalin and topiramate, exposure was usually limited to the 1st trimester of pregnancy (Supplementary Table 3). Children included in the cohort were followed until a mean age of 3.6 years (maximum: 5 years) (Supplementary Table 3).

A total of 15,270 children (0.9%) were identified with NDs during follow-up, mainly based on a diagnosis of the “disorders of psychological development” category (10,010 cases, 0.6%), including 4280 cases (0.3%) of “pervasive developmental disorders”; and, to a lesser degree, a diagnosis of the “behavioural and emotional disorders with onset usually occurring in childhood and adolescence” (4398 cases, 0.3%) or “mental retardation” (3398 cases, 0.2%) categories. The majority of diagnoses (62%) were derived from hospitalisation data (Supplementary Table 4). A total of 72,498 children (4.2%) utilized speech therapy services, 204,693 (11.9%) utilized orthoptic services and 22,539 (1.3%) utilized psychiatric services (Supplementary Table 5).

### Exposure to VPA

Mothers of children exposed to VPA were older, more socioeconomically deprived, more often received folic acid supplementation, more often users of alcohol or tobacco, antidepressants, antipsychotics, anxiolytics and hypnotics (both before and during pregnancy) than mothers of unexposed children. Children exposed to VPA were more often born preterm, with a birth weight less than 2500 g and were small for gestational age (Table 1). These differences tended to be less pronounced or tended to disappear in children born to a mother with no known mental illness (Supplementary Table 6).

**Table 1:** Maternal and neonatal characteristics by exposure group—overall study population. N (%) are shown.

		Unexposed*	Exposed to valproate	Exposed to lamotrigine	Exposed to carbamazepine	Exposed to clonazepam	Exposed to gabapentin	Exposed to levetiracetam	Exposed to oxcarbazepine	Exposed to phenobarbital	Exposed to pregabalin	Exposed to topiramate
		(N = 1,710,441)	(N = 991)	(N = 2,813)	(N = 468)	(N = 1,246)	(N = 378)	(N = 621)	(N = 143)	(N = 84)	(N = 1,627)	(N = 477)
**Maternal characteristics**												
Age at the end of pregnancy (years)	Mean (standard deviation)	29.8 (5.3)	31.0 (5.7)	29.9 (5.1)	31.7 (5.6)	32.1 (5.5)	32.2 (5.5)	29.3 (5.3)	30.6 (5.7)	31.4 (6.6)	32.3 (5.7)	30.5 (5.3)
	< 25	276,843 (16.2)	137 (13.8)	424 (15.1)	50 (10.7)	118 (9.5)	32 (8.5)	119 (19.2)	23 (16.1)	18 (21.4)	139 (8.5)	66 (13.8)
	[25–30]	562,803 (32.9)	259 (26.1)	916 (32.6)	111 (23.7)	302 (24.2)	90 (23.8)	212 (34.1)	32 (22.4)	15 (17.9)	414 (25.4)	144 (30.2)
	[30–35]	547,199 (32.0)	310 (31.3)	942 (33.5)	156 (33.3)	380 (30.5)	125 (33.1)	181 (29.1)	52 (36.4)	20 (23.8)	495 (30.4)	153 (32.1)
	≥ 35	323,596 (18.9)	285 (28.8)	531 (18.9)	151 (32.3)	446 (35.8)	131 (34.7)	109 (17.6)	36 (25.2)	31 (36.9)	579 (35.6)	114 (23.9)
Complementary Universal Health Insurance scheme		270,199 (15.8)	311 (31.4)	446 (15.9)	92 (19.7)	303 (24.3)	82 (21.7)	134 (21.6)	36 (25.2)	27 (32.1)	404 (24.8)	79 (16.6)
Salary reconstituted from the sum of maternity leave allowances	Missing data	660,198 (38.6)	552 (55.7)	1,157 (41.1)	218 (46.6)	577 (46.3)	162 (42.9)	264 (42.5)	67 (46.9)	50 (59.5)	701 (43.1)	190 (39.8)
	< €1000 **	75,800 (7.2)	58 (13.2)	161 (9.7)	32 (12.8)	58 (8.7)	17 (7.9)	43 (12.0)	7 (9.2)	6 (17.6)	117 (12.6)	27 (9.4)
	€1000–1499 **	156,470 (14.9)	102 (23.2)	256 (15.5)	43 (17.2)	126 (18.8)	50 (23.1)	73 (20.4)	18 (23.7)	9 (26.5)	177 (19.1)	63 (22.0)
	€1500–1999 **	349,119 (33.2)	144 (32.8)	612 (37.0)	91 (36.4)	216 (32.3)	81 (37.5)	118 (33.1)	26 (34.2)	10 (29.4)	366 (39.5)	103 (35.9)
	€2000–2499 **	222,451 (21.2)	74 (16.9)	310 (18.7)	53 (21.2)	135 (20.2)	34 (15.7)	56 (15.7)	17 (22.4)	7 (20.6)	136 (14.7)	61 (21.3)
	€2500–2999 **	118,169 (11.3)	38 (8.7)	160 (9.7)	19 (7.6)	48 (7.2)	19 (8.8)	37 (10.4)	5 (6.6)	1 (2.9)	74 (8.0)	16 (5.6)
	≥ €3000 **	128,234 (12.2)	23 (5.2)	157 (9.5)	12 (4.8)	86 (12.9)	15 (6.9)	30 (8.4)	3 (3.9)	1 (2.9)	56 (6.0)	17 (5.9)
Year of end of pregnancy	2011	321,046 (18.8)	243 (24.5)	490 (17.4)	121 (25.9)	601 (48.2)	59 (15.6)	90 (14.5)	32 (22.4)	15 (17.9)	230 (14.1)	93 (19.5)
	2012	488,371 (28.6)	333 (33.6)	778 (27.7)	134 (28.6)	500 (40.1)	90 (23.8)	147 (23.7)	50 (35.0)	32 (38.1)	412 (25.3)	118 (24.7)
	2013	471,047 (27.5)	233 (23.5)	793 (28.2)	134 (28.6)	83 (6.7)	116 (30.7)	202 (32.5)	27 (18.9)	23 (27.4)	465 (28.6)	128 (26.8)
	2014	429,977 (25.1)	182 (18.4)	752 (26.7)	79 (16.9)	62 (5.0)	113 (29.9)	182 (29.3)	34 (23.8)	14 (16.7)	520 (32.0)	138 (28.9)
Folic acid		482,209 (28.2)	525 (53.0)	1,978 (70.3)	268 (57.3)	360 (28.9)	139 (36.8)	396 (63.8)	89 (62.2)	32 (38.1)	440 (27.0)	179 (37.5)
Indicator of alcohol use		5,503 (0.3)	22 (2.2)	27 (1.0)	2 (0.4)	35 (2.8)	6 (1.6)	13 (2.1)	1 (0.7)	2 (2.4)	21 (1.3)	7 (1.5)
Indicator of tobacco use		137,968 (8.1)	136 (13.7)	283 (10.1)	53 (11.3)	165 (13.2)	66 (17.5)	69 (11.1)	13 (9.1)	8 (9.5)	257 (15.8)	43 (9.0)
Diagnosis of mental illness other than tobacco and alcohol use disorders		41,553 (2.4)	96 (9.7)	287 (10.2)	74 (15.8)	216 (17.3)	43 (11.4)	51 (8.2)	36 (25.2)	15 (17.9)	169 (10.4)	54 (11.3)
Antidepressants before pregnancy		105,848 (6.2)	107 (10.8)	399 (14.2)	84 (17.9)	494 (39.6)	143 (37.8)	68 (11.0)	37 (25.9)	13 (15.5)	603 (37.1)	185 (38.8)
Antidepressants during pregnancy		38,561 (2.3)	58 (5.9)	239 (8.5)	67 (14.3)	393 (31.5)	100 (26.5)	30 (4.8)	29 (20.3)	7 (8.3)	422 (25.9)	113 (23.7)
SSRI during pregnancy		27,188 (1.6)	33 (3.3)	168 (6.0)	36 (7.7)	201 (16.1)	42 (11.1)	23 (3.7)	22 (15.4)	5 (6.0)	172 (10.6)	55 (11.5)
Antipsychotics before pregnancy		12,497 (0.7)	49 (4.9)	131 (4.7)	32 (6.8)	123 (9.9)	24 (6.3)	15 (2.4)	31 (21.7)	6 (7.1)	79 (4.9)	30 (6.3)
Antipsychotics during pregnancy		11,281 (0.7)	37 (3.7)	77 (2.7)	24 (5.1)	109 (8.7)	17 (4.5)	8 (1.3)	24 (16.8)	5 (6.0)	67 (4.1)	27 (5.7)
Anxiolytics before pregnancy		243,326 (14.2)	284 (28.7)	980 (34.8)	176 (37.6)	522 (41.9)	150 (39.7)	232 (37.4)	59 (41.3)	28 (33.3)	659 (40.5)	188 (39.4)
Anxiolytics during pregnancy		106,177 (6.2)	206 (20.8)	675 (24.0)	120 (25.6)	389 (31.2)	112 (29.6)	173 (27.9)	50 (35.0)	23 (27.4)	429 (26.4)	103 (21.6)
Hypnotics before pregnancy		69,084 (4.0)	93 (9.4)	246 (8.7)	44 (9.4)	287 (23.0)	72 (19.0)	57 (9.2)	20 (14.0)	12 (14.3)	315 (19.4)	71 (14.9)
Hypnotics during pregnancy		18,314 (1.1)	46 (4.6)	94 (3.3)	28 (6.0)	157 (12.6)	32 (8.5)	28 (4.5)	15 (10.5)	4 (4.8)	149 (9.2)	34 (7.1)
Indicator of severity of psychiatric morbidity (number of ATC classes of psychotropic drugs)	0	1,406,243 (82.2)	653 (65.9)	1,640 (58.3)	263 (56.2)	492 (39.5)	157 (41.5)	366 (58.9)	65 (45.5)	53 (63.1)	701 (43.1)	217 (45.5)
	1	184,570 (10.8)	180 (18.2)	658 (23.4)	107 (22.9)	283 (22.7)	91 (24.1)	138 (22.2)	33 (23.1)	10 (11.9)	355 (21.8)	92 (19.3)
	2	67,558 (3.9)	67 (6.8)	247 (8.8)	34 (7.3)	149 (12.0)	49 (13.0)	54 (8.7)	14 (9.8)	8 (9.5)	227 (14.0)	69 (14.5)
	3	27,762 (1.6)	28 (2.8)	106 (3.8)	26 (5.6)	102 (8.2)	40 (10.6)	28 (4.5)	5 (3.5)	4 (4.8)	133 (8.2)	36 (7.5)
	4	12,338 (0.7)	17 (1.7)	71 (2.5)	11 (2.4)	81 (6.5)	14 (3.7)	11 (1.8)	4 (2.8)	5 (6.0)	83 (5.1)	32 (6.7)
	≥ 5	11,970 (0.7)	46 (4.6)	91 (3.4)	27 (5.8)	139 (11.2)	27 (7.2)	24 (3.9)	22 (15.4)	4 (4.8)	128 (7.9)	31 (6.5)
**Neonatal characteristics**												
Gestational age at birth	Mean (standard deviation)	39.1 (1.7)	39.0 (1.8)	39.0 (1.7)	39.1 (1.8)	38.9 (1.9)	38.6 (2.1)	38.9 (2.0)	38.8 (1.9)	38.8 (2.0)	38.9 (1.9)	39.0 (2.1)
	22–26 WA	2041 (0.1)	1 (0.1)	2 (0.1)	0 (0.0)	1 (0.1)	0 (0.0)	1 (0.2)	1 (0.7)	0 (0.0)	3 (0.2)	1 (0.2)
	27–31 WA	8584 (0.5)	6 (0.6)	9 (0.3)	3 (0.6)	9 (0.7)	2 (0.5)	6 (1.0)	0 (0.0)	1 (1.2)	9 (0.6)	6 (1.3)
	32–34 WA	20,638 (1.2)	19 (1.9)	40 (1.4)	7 (1.5)	28 (2.2)	23 (6.1)	11 (1.8)	3 (2.1)	1 (1.2)	37 (2.3)	10 (2.1)
	35–36 WA	59,992 (3.5)	45 (4.5)	124 (4.4)	12 (2.6)	55 (4.4)	21 (5.6)	33 (5.3)	6 (4.2)	3 (3.6)	61 (3.7)	19 (4.0)
	≥ 37 WA	1,619,186 (94.7)	920 (92.8)	2638 (93.8)	446 (95.3)	1153 (92.5)	332 (87.8)	570 (91.8)	133 (93.0)	79 (94.0)	1517 (93.2)	441 (92.5)
Sex	Male	873,972 (51.1)	496 (50.1)	1471 (52.3)	243 (51.9)	597 (47.9)	206 (54.5)	292 (47.0)	65 (45.5)	43 (51.2)	847 (52.1)	254 (53.2)
Weight at birth	Mean (standard deviation)	3,288 (513)	3,209 (550)	3,262 (515)	3,274 (542)	3,220 (553)	3,190 (600)	3,152 (540)	3,218 (597)	3,087 (593)	3,263 (563)	3,257 (579)
	< 2500 g	91,455 (5.3)	88 (8.9)	172 (6.1)	31 (6.6)	105 (8.4)	47 (12.4)	60 (9.7)	12 (8.4)	12 (14.3)	118 (7.3)	32 (6.7)
	2500–2999 g	337,541 (19.7)	227 (22.9)	606 (21.5)	103 (22.0)	278 (22.3)	73 (19.3)	152 (24.5)	30 (21.0)	20 (23.8)	324 (19.9)	90 (18.9)
	3000–3499 g	704,348 (41.2)	389 (39.3)	1,156 (41.1)	176 (37.6)	485 (38.9)	140 (37.0)	251 (40.4)	60 (42.0)	35 (41.7)	630 (38.7)	203 (42.6)
	≥ 3500 g	577,097 (33.7)	287 (29.0)	879 (31.2)	158 (33.8)	378 (30.3)	118 (31.2)	158 (25.4)	41 (28.7)	17 (20.2)	555 (34.1)	152 (31.9)
Small for gestational age		167,376 (9.8)	149 (15.0)	284 (10.1)	48 (10.3)	159 (12.8)	45 (11.9)	82 (13.2)	14 (9.8)	17 (20.2)	177 (10.9)	55 (11.5)
Neonatal condition		222,201 (13.0)	119 (12.0)	349 (12.4)	59 (12.6)	197 (15.8)	53 (14.0)	85 (13.7)	17 (11.9)	9 (10.7)	231 (14.2)	74 (15.5)

*Not exposed to any antiepileptic drug during pregnancy. ** among those for whom the information was available.

In multivariable analysis, children exposed to VPA, compared to unexposed children, were at higher risk of NDs (aHR, 3.7; 95% CI 2.8–4.9)—especially pervasive developmental disorders (aHR, 4.6; 95% CI 2.9–7.5), disorders of psychological development (aHR, 4.7; 95% CI 3.5–6.4) and mental retardation (aHR, 5.1; 95% CI 3.1–8.5)—and utilization of speech therapy (aHR, 1.7; 95% CI 1.4–2.1). These estimates were similar or higher among children born to a mother with no known mental illness (Table 2). Comparison with children exposed to lamotrigine showed three to fourfold increased risks of pervasive developmental disorders, mental retardation and disorders of psychological development with VPA (Supplementary Table 7).

**Table 2:** Incidence of early neurodevelopmental disorders during follow-up according to exposure to valproate during pregnancy (compared to unexposed children) in the overall study population and among children born to a mother with no known mental illness.

	Overall population					Children born to a mother with no known mental illness				
	Children exposed to valproate during pregnancy (N = 991)		Unexposed children (N = 1,710,441)		aHR [95%CI]*	Children exposed to valproate during pregnancy (N = 619)		Unexposed children (N = 1,382,288)		aHR [95%CI]*
	N	IR per 1,000 PY	N	IR per 1,000 PY		N	IR per 1,000 PY	N	IR per 1,000 PY	
**Diagnosis**										
Mental and behavioural disorders (F70–F98)	50	13.5	15,270	2.5	**3.7 [2.8–4.9]**	31	13.4	11,012	2.2	**5.1 [3.6–7.3]**
Pervasive developmental disorders (F84)	17	4.5	4,280	0.7	**4.6 [2.9–7.5]**	11	4.7	3,131	0.6	**6.4 [3.5–11.5]**
Mental retardation (F70–F79)	15	4.0	3,398	0.6	**5.1 [3.1–8.5]**	8	3.4	2,544	0.5	**5.5 [2.7–11.0]**
Disorders of psychological development (F80–F89)	41	11.0	10,010	1.6	**4.7 [3.5–6.4]**	28	12.1	7,315	1.5	**6.9 [4.8–10.0]**
Behavioural and emotional disorders with onset usually occurring in childhood and adolescence (F90–F98)	7	1.8	4,398	0.7	1.7 [0.8–3.5]	4	1.7	2,976	0.6	2.4 [0.9–6.3]
**Health care utilization**										
Speech therapy	93	25.1	72,012	11.9	**1.7 [1.4–2.1]**	62	27.0	55,532	11.3	**2.1 [1.6–2.7]**
Orthoptics	135	38.4	203,489	35.6	1.1 [0.9–1.3]	79	36.0	162,427	35.3	1.0 [0.8–1.3]
Psychiatry	22	5.8	22,365	3.7	1.2 [0.8–1.9]	12	5.1	16,018	3.3	1.4 [0.8–2.5]

Bold values indicate statistically significant associations, i.e. with a p-value ≤ 0.05.

*IR* Incidence rate, *HR* Hazard ratio, *95%CI* 95% confidence interval, *PY* person-year.

*Cox models adjusted for: mother’s age, Complementary Universal Health Insurance scheme, diagnosis of mental illness other than tobacco and alcohol use disorders, antipsychotic drug use during the year preceding pregnancy, indicator of severity of psychiatric morbidity, indicator of tobacco use, indicator of alcohol use, folic acid, SSRI during pregnancy, child’s sex, gestational age and birth weight.

The risk of NDs and health care utilization during follow-up among children exposed to VPA differed according to the period of in utero exposure (Table 3). Prolonged exposures during pregnancy (1st trimester and at least during the 2nd or 3rd trimester) were associated with higher risks, whereas exposure exclusively during the 1st trimester of pregnancy was not associated with an increased risk of any of the diagnostic categories or health care utilizations studied (but only a limited number of cases were observed). Highest risks were also observed with highest doses of VPA, but the risks of mental retardation and disorders of psychological development were increased even in the lower tertiles of VPA doses (Supplementary Table 8).

**Table 3:** Incidence of early neurodevelopmental disorders during follow-up according to the period of exposure to VPA during pregnancy in the overall study population (compared to unexposed children).

	Unexposed children (N = 1,710,441)		Children exposed to valproate only during the 1st trimester of pregnancy (N = 232)			Children exposed to valproate during the 1st trimester and the 2nd or 3rd trimester of pregnancy (N = 601)			Children exposed to valproate only during the 2nd or 3rd trimester of pregnancy (N = 158)		
	N	IR per 1,000 PY	N	IR per 1,000 PY	aHR [95%CI]*	N	IR per 1,000 PY	aHR [95%CI]*	N	IR per 1,000 PY	aHR [95%CI]*
**Diagnosis**											
Mental and behavioural disorders (F70–F98)	15,270	2.5	4	4.6	1.0 [0.4–2.6]	40	17.7	**5.5 [4.0–7.4]**	6	10.1	**3.0 [1.3–6.6]**
Pervasive developmental disorders (F84)	4,280	0.7	1	1.1	0.9 [0.1–6.7]	14	6.1	**7.0 [4.1–11.8]**	2	3.3	3.4 [0.8–13.4]
Mental retardation (F70–F79)	3,398	0.6	2	2.3	2.5 [0.6–9.9]	10	4.3	**6.0 [3.2–11.1]**	3	5.0	**6.8 [2.2–21.1]**
Disorders of psychological development (F80–F89)	10,010	1.6	2	2.3	0.8 [0.2–3.1]	33	14.5	**6.9 [4.9–9.7]**	6	10.0	**4.4 [2.0–9.9]**
Behavioural and emotional disorders with onset usually occurring in childhood and adolescence (F90–F98)	4,398	0.7	2	2.3	1.5 [0.4–5.9]	5	2.2	2.2 [0.9–5.3]	0	0.0	-
**Health care utilization**											
Speech therapy	72,012	11.9	17	19.9	1.3 [0.8–2.0]	70	31.3	**2.2 [1.8–2.8]**	6	9.9	0.7 [0.3–1.5]
Orthoptics	203,489	35.6	24	29.3	0.8 [0.5–1.2]	98	46.5	**1.3 [1.0–1.5]**^**a**^	13	22.2	0.7 [0.4–1.2]
Psychiatry	22,365	3.7	5	5.8	1.2 [0.5–2.8]	14	6.0	1.3 [0.8–2.2]	3	5.0	1.2 [0.4–3.8]

Bold values indicate statistically significant associations, i.e. with a p-value ≤ 0.05.

*IR* Incidence rate, *HR* Hazard ratio, *95%CI* 95% confidence interval.

*Cox models adjusted for: mother’s age, Complementary Universal Health Insurance scheme, diagnosis of mental illness other than tobacco and alcohol use disorders, antipsychotics during the year preceding pregnancy, indicator of severity of psychiatric morbidity, indicator of tobacco use, indicator of alcohol use, folic acid, SSRI during pregnancy, child’s sex, gestational age and birth weight.

^a^p = 0.03.

### Exposure to lamotrigine and carbamazepine

As for VPA, mothers exposed to lamotrigine and especially carbamazepine were more socioeconomically deprived, more often users of alcohol or tobacco, psycholeptics and psychoanaleptics, their children more often had a lower birth weight and were more often small for gestational age (Table 1); and these differences were less pronounced (carbamazepine) or disappeared (lamotrigine) in children born to a mother with no known mental illness (Supplementary Table 6).

In multivariable analysis, children exposed to lamotrigine, compared to unexposed children, were at higher risk of NDs (aHR, 1.6; 95% CI 1.2–2.1), especially disorders of psychological development (aHR, 1.5; 95% CI 1.0–2.1) and mental retardation (aHR, 2.4; 95% CI 1.4–4.0). These estimates slightly decreased and were no longer significant among children born to a mother with no known mental illness, except for utilization of speech therapy (aHR, 1.3; 95% CI 1.0–1.6) (Table 4). Children exposed to carbamazepine, compared to unexposed children, were at higher risk of NDs (aHR, 1.9; 95% CI 1.0–3.4), especially disorders of psychological development (aHR, 2.0; 95% CI 1.0–4.1) and behavioural and emotional disorders retardation (aHR, 3.2; 95% CI 1.4–7.1) (Supplementary Table 9). There was no increased risk for any of the outcomes among children born to a mother with no known mental illness.

**Table 4:** Incidence of early neurodevelopmental disorders during follow-up according to exposure to lamotrigine during pregnancy (compared to unexposed children) in the overall study population and among children born to a mother with no known mental illness.

	Overall population					Children born to a mother with no known mental illness				
	Children exposed to lamotrigine during pregnancy (N = 2,813)		Unexposed children (N = 1,707,707)		aHR [95%CI]*	Children exposed to lamotrigine during pregnancy (N = 1,586)		Unexposed children (N = 1,382,176)		aHR [95%CI]**
	N	IR per 1,000 PY	N	IR per 1,000 PY		N	IR per 1,000 PY	N	IR per 1,000 PY	
**Diagnosis**										
Mental and behavioural disorders (F70–F98)	47	4.7	15,165	2.5	**1.6 [1.2–2.1]**	17	3.0	11,010	2.2	1.4 [0.9–2.2]
Pervasive developmental disorders (F84)	10	1.0	4,254	0.7	1.3 [0.7–2.4]	5	0.9	3,131	0.6	1.4 [0.6–3.5]
Mental retardation (F70–F79)	15	1.5	3,385	0.6	**2.4 [1.4–4.0]**	4	0.7	2,544	0.5	1.4 [0.5–3.8]
Disorders of psychological development (F80–F89)	27	2.7	9,949	1.6	**1.5 [1.0–2.1]**^**a**^	12	2.1	7,315	1.5	1.5 [0.8–2.6]
Behavioural and emotional disorders with onset usually occurring in childhood and adolescence (F90–F98)	10	1.0	4,352	0.7	1.0 [0.6–1.9]	1	0.2	2,974	0.6	0.3 [0.0–2.1]
**Health care utilization**										
Speech therapy	149	14.9	71,832	11.8	1.2 [1.0–1.4]^b^	85	15.1	55,527	11.3	**1.3 [1.0–1.6]**^**d**^
Orthoptics	410	44.1	203,120	35.6	**1.1 [1.0–1.2]**^**c**^	214	40.5	162,414	35.3	1.1 [0.9–1.2]
Psychiatry	49	4.9	22,261	3.6	1.0 [0.7–1.3]	17	3.0	16,015	3.3	0.8 [0.5–1.3]

Bold values indicate statistically significant associations, i.e. with a p-value ≤ 0.05.

*IR* Incidence rate, *HR* Hazard ratio, *95%CI* 95% confidence interval.

*Cox models adjusted for: mother’s age, Complementary Universal Health Insurance scheme, diagnosis of mental illness other than tobacco and alcohol use disorders, antipsychotic drug use during the year preceding pregnancy, indicator of severity of psychiatric morbidity, indicator of tobacco use, indicator of alcohol use, folic acid, SSRI during pregnancy, child’s sex, gestational age and birth weight.

**Cox models adjusted for: mother’s age, Complementary Universal Health Insurance scheme, indicator of tobacco use, indicator of alcohol use, folic acid, child’s sex, gestational age and birth weight.

^a^p = 0.05, ^b^p = 0.07, ^c^p = 0.02, ^d^p = 0.03.

### Exposure to other AEDs not indicated in bipolar disorder

Mothers of children exposed to other AED were generally older (except for levetiracetam), more socioeconomically deprived (particularly those using phenobarbital), and more often users of alcohol, tobacco antidepressants, antipsychotics, anxiolytics and hypnotics than mothers of unexposed children. However, no marked differences in terms of any of the child characteristics was observed between the two groups (Table 1 and Supplementary Table 6).

The risks of NDs and health care utilization during follow-up associated with these AEDs are presented in Table 5 (pregabalin) and in Supplementary Tables 10–15 (other AEDs). No consistent association was found for these AEDs, except for pregabalin, which was associated with a 1.5-fold increased risk of NDs (aHR: 1.5, 95% CI 1.0 to 2.1) with, in particular, an increased risk of mental retardation (aHR: 3.1, 95% CI 1.2 to 8.3) and utilization of orthoptic services in the absence of maternal mental illness; and phenobarbital, which was associated with a sevenfold higher risk of a diagnosis of “behavioural and emotional disorders with onset usually occurring in childhood and adolescence” among children born to a mother with no known mental illness (aHR: 7.6, 95% CI 1.1 to 53.6). However, in utero exposures to levetiracetam, oxcarbazepine or topiramate were associated with increased health care utilization (orthoptics for levetiracetam and oxcarbazepine, speech therapy for topiramate and psychiatry for levetiracetam).

**Table 5:** Incidence of early neurodevelopmental disorders during follow-up according to exposure to pregabalin during pregnancy (compared to unexposed children) in the overall study population and among children born to a mother with no known mental illness.

	Overall population					Children born to a mother with no known mental illness				
	Children exposed to pregabalin during pregnancy (N = 1,627)		Unexposed children (N = 1,710,441)		aHR [95%CI]*	Children exposed to pregabalin during pregnancy (N = 674)		Unexposed children (N = 1,382,288)		aHR [95%CI]**
	N	IR per 1,000 PY	N	IR per 1,000 PY		N	IR per 1,000 PY	N	IR per 1,000 PY	
**Diagnosis**										
Mental and behavioural disorders (F70–F98)	28	5.0	15,270	2.5	**1.5 [1.0–2.1]**^**a**^	7	3.1	11,012	2.2	1.3 [0.6–2.7]
Pervasive developmental disorders (F84)	7	1.2	4,280	0.7	1.4 [0.7–2.9]	1	0.4	3,131	0.6	0.7 [0.1–4.8]
Mental retardation (F70–F79)	7	1.2	3,398	0.6	1.7 [0.8–3.6]	4	1.7	2,544	0.5	**3.1 [1.2–8.3]**
Disorders of psychological development (F80–F89)	16	2.9	10,010	1.6	1.3 [0.8–2.2]	4	1.7	7,315	1.5	1.1 [0.4–3.0]
Behavioural and emotional disorders with onset usually occurring in childhood and adolescence (F90–F98)	9	1.6	4,398	0.7	1.4 [0.8–2.8]	2	0.9	2,976	0.6	1.4 [0.3–5.5]
**Health care utilization**										
Speech therapy	61	11.0	72,012	11.9	0.9 [0.7–1.2]	24	10.5	55,532	11.3	1.0 [0.7–1.5]
Orthoptics	225	43.3	203,489	35.6	**1.2 [1.1–1.4]**	98	46.0	162,427	35.3	**1.4 [1.1–1.6]**
Psychiatry	23	4.1	22,365	3.7	0.9 [0.6–1.3]	7	3.1	16,018	3.3	1.0 [0.5–2.1]

Bold values indicate statistically significant associations, i.e. with a p-value ≤ 0.05.

*IR* Incidence rate, *HR* hazard ratio, *95% CI* 95% confidence interval.

*Cox models adjusted for: mother’s age, Complementary Universal Health Insurance scheme, diagnosis of mental illness other than tobacco and alcohol use disorders, antipsychotics during the year preceding pregnancy, indicator of severity of psychiatric morbidity, indicator of tobacco use, indicator of alcohol use, folic acid, SSRI during pregnancy, child’s sex, gestational age and birth weight.

**Cox models adjusted for: mother’s age, Complementary Universal Health Insurance scheme, indicator of tobacco use, indicator of alcohol use, folic acid, child’s sex, gestational age and birth weight.

^a^p = 0.04.

## Discussion

This large nationwide cohort study provides new information on the risks of early NDs associated with in utero exposure to VPA and the other main AEDs currently used. Firstly, it shows a four to fivefold higher risk of early NDs following exposure to VPA, more specifically concerning pervasive developmental disorders, mental retardation and disorders of psychological development. Exposure to VPA, which was found to have a dose–response relationship with occurrence of NDs, also had a different impact according to the period of exposure: children exposed during the second and/or third trimesters of pregnancy had a markedly increased risk of early NDs, unlike children exposed to VPA only during the first trimester. Secondly, the risk associated with other AEDs appeared to be much lower than that associated with VPA. The slight increases in the risk of several mental and behavioural disorders with lamotrigine and carbamazepine were no longer observed when the analysis was confined to children born to a mother with no known mental illness, suggesting an effect of maternal mental illness or associated characteristics rather than exposure to these drugs. The associations observed between pregabalin and phenobarbital and mental retardation, behavioural and emotional disorders and utilization of orthoptic services were based on limited or very limited sample sizes. For the other AEDs, either no increased risk was observed (clonazepam or gabapentin) or only associations with health care utilizations services (oxcarbazepine, levetiracetam, topiramate), often at the limit of significance.

The results for VPA confirm published data concerning both the level of increased risk of NDs associated with exposure during pregnancy, the nature of these disorders and the dose-dependent nature of this risk. Several studies have demonstrated a five to sevenfold increased risk of autism spectrum disorders^10,16,19,23–25^, a four to eightfold increased risk of special educational needs^6,9^, and a two to threefold increased risk of language delay^11–13^ related to in utero exposure to VPA. Moreover, lower IQ, attention and memory scores have been reported among 6-year-old children exposed to VPA in utero^10^. Prenatal exposure to VPA has also been associated with an increased risk of attention-deficit/hyperactivity disorder^15^ and decreased sociability and communication scores^14,17^ and school performances in 6-year-old children^7^ and adolescents^8^. Previous studies have reported the existence of a dose–effect relationship^20,21^, and one study even suggested a threshold effect, with no observed effect for doses lower than 800 mg/day^9^. In contrast, in our study the risks of NDs were increased even at low VPA doses. Experimental data suggest that VPA, which acts on GABA signalling, induces hippocampal and cortical dysplasia^26^.

This study also provides new data concerning the heterogeneous level of risk of early NDs according to the period of exposure to VPA during pregnancy. Although some studies have suggested that the increased risk of NDs is predominantly observed following exposure during the third trimester^27,28^, others have reported harmful effects regardless of the period of exposure during pregnancy^29^. Our results suggest that the exposure period associated with an increased risk of early NDs essentially corresponds to the second and/or third trimesters of pregnancy, although no definitive conclusions can be drawn due to the limited number of cases.

Our results concerning lamotrigine support published data, generally suggesting no impact of exposure to lamotrigine during pregnancy on the risks of NDs, particularly on the risk of mental retardation^18,20,21^. However, some studies have suggested an impact of in utero exposure to lamotrigine on the risk of impaired motor and sensory capacities^30^, disorders of eye-hand coordination^31^, or language delay or onset of autistic trait^13^, and experimental data are not completely reassuring^32^. Longer-term follow-up of our cohort may enable us to more reliably detect the potential effects of lamotrigine on these disorders.

Regarding carbamazepine, phenobarbital and topiramate, although experimental studies have not demonstrated any effects^26,32^, increased risks of cognitive and motor disorders have been reported in children exposed prenatally^9,10,13,15,27,33–35^. Our results suggest a possible increased risk of early NDs associated with exposure to carbamazepine and phenobarbital but not with topiramate. However, these results were based on limited numbers of cases among exposed children and should be interpreted with caution.

Almost no data concerning the risks of NDs associated with the other AEDs considered in our study are available in the literature. The results of the present study do not suggest an increased risk of a diagnosis of NDs associated with exposure to clonazepam, gabapentin, levetiracetam or oxcarbazepine during pregnancy. However, our finding of an increased risk of mental retardation and utilization of orthoptic services among children exposed to pregabalin constitutes a signal that needs to be further investigated.

### Strengths and limitations

This study, based on the largest cohort of children exposed to AEDs in utero, constitutes a major source of information on the risk of NDs associated with prenatal exposure to these drugs. Up until now, studies on this risk have generally been limited to a small number of drugs and much smaller sample sizes. Linking the mother and child data from the national SNDS databases allowed this study to be conducted on a large unselected population, for which exposure to AEDs was measured without memory bias, in contrast with previous studies generally based on retrospective exposure data derived from registries. The data available in the SNDS also allowed the analysis of various indicators of NDs based on diagnoses or health care utilization. Finally, the available information allowed to account for differences in terms of sociodemographic characteristics, maternal health status and neonatal characteristics according to exposure to the various AEDs considered.

However, this study comprises some limitations. Firstly, as identification of NDs was based on diagnoses established during a hospitalisation or for management of a LTD, or utilization of speech therapy, orthoptic or psychiatric services, any case not associated with this type of event would not be identified. Furthermore, the limited follow-up in the study (up until a mean age of 3.6 years) probably resulted in identifying only the most severe cases, while less severe cases would need a longer follow-up to be detected. These limitations would have led to underestimate the incidence of NDs, especially those that typically manifest at later stages of development (eg, learning disorders or ADHD), and this underestimation may differ according to the exposure group, as the time to diagnosis could be shorter among children exposed in utero to VPA due to closer follow-up. Furthermore, due to the lack of specificity of the indicators of health care utilization, results based on these indicators must be interpreted cautiously.

Moreover, due to the observational nature of the data, reported differences according to AED exposure must be interpreted cautiously. Differences between children exposed to each drug *versus* those not exposed to any AED during pregnancy could partly reflect an effect of maternal epilepsy on the risk of NDs in the offspring. However, previous studies suggest that such an effect, if any, would be very limited^9,13,31,36^. In addition, the increased risk of NDs in children exposed to VPA persisted even when compared to children exposed to lamotrigine. Women with genetic epilepsy syndromes (also having genetic vulnerability to NDs) are often prescribed valproate as first line therapy; however, it is unlikely that these syndromes were common enough to explain the large (four to fivefold) increase in risk of NDs associated with VPA observed in this study.

Maternal psychiatric morbidity and comedications, which constitute major risk factors for NDs^37^, could also explain the observed differences between AED exposure groups. However, maternal psychiatric morbidity and comedications were accounted for in the analyses using information available in the SNDS. In addition, analyses were systematically conducted both in the overall population and in the subpopulation of children born to a mother with no known mental illness. The increased risk of NDs associated with VPA persisted or was even amplified in this restricted analysis, suggesting that the observed association cannot be explained by differences in terms of maternal psychiatric morbidity.

Information available in the SNDS on folic acid supplementation is probably subject to measurement errors since dispensing of folic acid is not always reimbursed by national health insurance. Thus, analyses may have failed to fully account for the putative protective effect^10,38–41^ of folic acid supplementation on the risk of NDs. Other potential confounding factors, such as characteristics of maternal epilepsy or parental IQ, could not be taken into account, as this information is not available in the SNDS.

Lastly, due to limited sample size and events in the group of women taking 200–800 mg/day of VPA, our study, which was restricted to AEDs used in monotherapy, was not powered to identify thresholds of risk within this range. Other studies would be necessary to document the safety of low doses of VPA as they may be currently used (in association with levetiracetam or lamotrigine for example) to limit the teratogenicity of VPA.

## Conclusions

This study confirms a four to fivefold increased risk of early NDs (before the age of 6 years), especially pervasive developmental disorders and mental retardation, associated with exposure to VPA indicated in epilepsy during pregnancy, with a marked dose effect. It also provides new data suggesting that the exposure period associated with an increased risk of early NDs is more particularly situated during the second and/or third trimesters of pregnancy. The risk of early NDs associated with other AEDs, especially lamotrigine, appears to be much lower. However, the risk of NDs after in utero exposure to pregabalin deserves further investigations.

## Supplementary information

Supplementary information

## Data Availability

Data cannot be shared publicly because access to French administrative medical databases is regulated by the French Data Protection Supervisory Authority (Commission Nationale de l'Informatique et des Libertés, CNIL). Data are available from the Commission Nationale de l'Informatique et des Libertés, CNIL (contact via https://www.cnil.fr/) for researchers who meet the criteria for access to confidential data.

## References

[1] Hauser WA, Annegers JF, Kurland LT (1993). Incidence of epilepsy and unprovoked seizures in Rochester, Minnesota: 1935–1984. Epilepsia.

[2] MacDonald BK, Cockerell OC, Sander JW, Shorvon SD (2000). The incidence and lifetime prevalence of neurological disorders in a prospective community-based study in the UK. Brain.

[3] Viale L, Allotey J, Cheong-See F (2015). Epilepsy in pregnancy and reproductive outcomes: A systematic review and meta-analysis. Lancet.

[4] Tomson T, Battino D, Bonizzoni E (2018). Comparative risk of major congenital malformations with eight different antiepileptic drugs: a prospective cohort study of the EURAP registry. Lancet Neurol..

[5] Blotière PO, Raguideau F, Weill A (2019). Risks of 23 specific malformations associated with prenatal exposure to ten antiepileptic drugs. Neurology.

[6] Adab N, Jacoby A, Smith D, Chadwick D (2001). Additional educational needs in children born to a mother with epilepsy. J. Neurol. Neurosurg. Psychiatry.

[7] Lacey AS, Pickrell WO, Thomas RH, Kerr MP, White CP, Rees M (2018). Educational attainment of children born to a mother with epilepsy. J. Neurol. Neurosurg. Psychiatry.

[8] Elkjær LS, Bech BH, Sun Y, Laursen TM, Christensen J (2018). Association between prenatal valproate exposure and performance on standardized language and mathematics tests in school-aged children. JAMA Neurol..

[9] Baker GA, Bromley RL, Briggs M (2015). IQ at 6 years after in utero exposure to antiepileptic drugs: A controlled cohort study. Neurology.

[10] Meador KJ, Baker GA, Browning N (2013). Fetal antiepileptic drug exposure and cognitive outcomes at age 6 years (NEAD study): A prospective observational study. Lancet Neurol..

[11] Nadebaum C, Anderson V, Vajda F, Reutens DC, Barton S, Wood AG (2011). Language skills of school-aged children prenatally exposed to antiepileptic drugs. Neurology.

[12] Dean JCS, Hailey H, Moore SJ, Lloyd DJ, Turnpenny PD, Little J (2002). Long term health and neurodevelopment in children exposed to antiepileptic drugs before birth. J. Med. Genet..

[13] Veiby G, Daltveit AK, Schjolberg S (2013). Exposure to antiepileptic drugs in utero and child development: A prospective population-based study. Epilepsia.

[14] Deshmukh U, Adams J, Macklin EA (2016). Behavioral outcomes in children exposed prenatally to lamotrigine, valproate, or carbamazepine. Neurotoxicol Teratol.

[15] Cohen MJ, Meador KJ, Browning N (2011). Fetal antiepileptic drug exposure: motor, adaptive, and emotional/behavioral functioning at age 3 years. Epilepsy Behav..

[16] Bromley RL, Mawer GE, Briggs M (2013). Liverpool and Manchester Neurodevelopment Group. The prevalence of neurodevelopmental disorders in children prenatally exposed to antiepileptic drugs. J Neurol Neurosurg Psychiatry.

[17] Vinten J, Bromley R, Taylor J, Adab N, Kini U, Baker GA (2009). Liverpool and Manchester Neurodevelopment Group. The behavioral consequences of exposure to antiepileptic drugs in utero. Epilepsy Behav..

[18] Veroniki AA, Rios P, Cogo E (2017). Comparative safety of antiepileptic drugs for neurological development in children exposed during pregnancy and breastfeeding: A systematic review and network meta-analysis. BMJ Open.

[19] Rasalam A, Hailey H, Williams J (2005). Characteristics of fetal anticonvulsant syndrome associated autistic disorder. Dev. Med. Child Neurol..

[20] Bromley RL, Weston J, Adab N (2014). Treatment for epilepsy in pregnancy: Neurodevelopmental outcomes in the child. Cochrane Database Syst Rev.

[21] Bromley RL, Baker GA (2017). Fetal antiepileptic drug exposure and cognitive outcomes. Seizure.

[22] Tuppin P, Rudant J, Constantinou P (2017). Value of a national administrative database to guide public decisions: From the système national d'information interrégimes de l'Assurance Maladie (SNIIRAM) to the système national des données de santé (SNDS) in France. Rev Epidemiol Sante Publique.

[23] Bromley RL, Mawer G, Clayton-Smith J, Baker GA (2008). Liverpool and Manchester Neurodevelopment Group Autism spectrum disorders following in utero exposure to antiepileptic drugs. Neurology.

[24] Cummings C, Stewart M, Stevenson M, Morrow J, Nelson J (2011). Neurodevelopment of children exposed in utero to lamotrigine, sodium valproate and carbamazepine. Arch. Dis. Child.

[25] Christensen J, Gronborg TK, Sorensen MJ (2013). Prenatal valproate exposure and risk of autism spectrum disorders and childhood autism. JAMA.

[26] Manent J-B, Jorquera I, Mazzucchelli I (2007). Fetal exposure to GABA-acting antiepileptic drugs generates hippocampal and cortical dysplasias. Epilepsia.

[27] Reinisch JM, Sanders WA, Mortensen EL, Rubin DB (1995). In utero exposure to phenobarbital and intelligence deficits in adult men. JAMA.

[28] Meador KJ, Baker G, Cohen MJ, Gaily E, Westerveld M (2007). Cognitive/behavioral teratogenetic effects of antiepileptic drugs. Epilepsy Behav..

[29] Harden CL, Meador KJ, Pennell PB (2009). Management issues for women with epilepsy-Focus on pregnancy (an evidence-based review): II. Teratogenesis and perinatal outcomes: Report of the Quality Standards Subcommittee and Therapeutics and Technology Subcommittee of the American Academy of Neurology and the American Epilepsy Society. Epilepsia.

[30] Rihtman T, Parush S, Ornoy A (2013). Developmental outcomes at preschool age after fetal exposure to valproic acid and lamotrigine: Cognitive, motor, sensory and behavioral function. Reprod Toxicol.

[31] Bromley RL, Mawer G, Love J (2010). Liverpool and Manchester Neurodevelopment Group Early cognitive development in children born to women with epilepsy: A prospective report. Epilepsia.

[32] Manent J-B, Jorquera I, Franco V, Ben-Ari Y, Perucca E, Represa A (2008). Antiepileptic drugs and brain maturation: fetal exposure to lamotrigine generates cortical malformations in rats. Epilepsy Res.

[33] Dessens AB, Cohen-Kettenis PT, Mellenbergh GJ, Koppe JG, van De Poll NE, Boer K (2000). Association of prenatal phenobarbital and phenytoin exposure with small head size at birth and with learning problems. Acta Paediatr.

[34] Rihtman T, Parush S, Ornoy A (2012). Preliminary findings of the developmental effects of in utero exposure to topiramate. Reprod Toxicol.

[35] Gopinath N, Muneer AK, Unnikrishnan S, Varma RP, Thomas SV (2015). Children (10–12 years age) of women with epilepsy have lower intelligence, attention and memory: observations from a prospective cohort case control study. Epilepsy Res.

[36] Gaily E, Kantola-Sorsa E, Hiilesmaa V (2004). Normal intelligence in children with prenatal exposure to carbamazepine. Neurology.

[37] Thorup AAE, Laursen TM, Munk-Olsen T et al, Incidence of child and adolescent mental disorders in children aged 0–17 with familial high risk for severe mental illness—A Danish register study. *Schizophr Res* 2017;pii:S0920–9964(17)30686–2. 10.1016/j.schres.2017.11.009 29132814

[38] Roth C, Magnus P, Schjølberg S (2011). Folic acid supplements in pregnancy and severe language delay in children. JAMA.

[39] Chatzi L, Papadopoulou E, Koutra K (2012). Effect of high doses of folic acid supplementation in early pregnancy on child neurodevelopment at 18 months of age: The mother–child cohort ‘Rhea’ study in Crete, Greece. Public Health Nutrition.

[40] Bjørk M, Riedel B, Spigset O (2018). Association of folic acid supplementation during pregnancy with the risk of autistic traits in children exposed to antiepileptic drugs in utero. JAMA Neurol..

[41] Levine SZ, Kodesh A, Viktorin A (2018). Association of maternal use of folic acid and multivitamin supplements in the periods before and during pregnancy with the risk of autism spectrum disorder in offspring. JAMA Psychiatry.

