# Supplementary material for: Risk of early neurodevelopmental disorders associated with in utero exposure to valproate and other antiepileptic drugs: a nationwide cohort study in France
Source: Sci Rep. 2020 Oct 22;10:17362. doi: 10.1038/s41598-020-74409-x (PMC7581762; doi:10.1038/s41598-020-74409-x)
Supplement: Supplementary file 1 — Supplementary information [file 41598_2020_74409_MOESM1_ESM.docx]

**Risk of early neurodevelopmental disorders associated with *in utero* exposure to valproate and other antiepileptic drugs: a nationwide cohort study in France**

Joël COSTE^1^, MD, PhD; Pierre-Olivier BLOTIERE^1,2^, PhD; Sara MIRANDA^3^, PharmD; Yann MIKAELOFF^4^, MD, PhD; Hugo PEYRE^5,6,7^, MD, PhD; Franck RAMUS^6^, PhD; Mahmoud ZUREIK^3,8^, MD, PhD; Alain WEILL^1^, MD; Rosemary DRAY-SPIRA^3^, MD, PhD (ORCID iD: https://orcid.org/0000-0001-7646-3667)

^1^ Department of Public Health Studies, French National Health Insurance (CNAM), Paris, France

^2^ Université de Lorraine, Université Paris-Descartes, Apemac, EA 4360, Nancy, France

^3^ Department of Epidemiology of Health Products, The French National Agency for Medicines and Health Products Safety (ANSM), Saint-Denis, France

^4^ Assistance Publique-Hôpitaux de Paris, Hôpital Bicêtre; INSERM, Université Paris-Saclay, 94805, Villejuif, France

^5^ Assistance Publique-Hôpitaux de Paris, Hôpital Robert Debré, Department of Child and Adolescent Psychiatry, Paris, France

^6^ Laboratoire de Sciences Cognitives et Psycholinguistique, Ecole Normale Supérieure, EHESS, CNRS, PSL University, Paris, France

^7^ INSERM UMR 1141, Paris Diderot University, Paris, France

^8^ Versailles Saint-Quentin University, Montigny le Bretonneux, France

**Appendix: Supplementary information**

Supplementary Table 1. Exposure to antiepileptic drugs during pregnancy

|  | **Monotherapy** | **Combination therapy** | **Total** |
| --- | --- | --- | --- |
| **Children not exposed to antiepileptic drugs** | **-** | **-** | 1,710,441 |
| **Children exposed to valproate** |  |  |  |
| Valproate | 991 | 500 | 1,491 |
| **Children exposed to drugs indicated for epilepsy and bipolar disorder** |  |  |  |
| Lamotrigine | 2,813 | 1,049 | 3,862 |
| Carbamazepine | 468 | 323 | 791 |
| **Children exposed to another antiepileptic drug** |  |  |  |
| Clonazepam | 1,246 | 313 | 1,559 |
| Gabapentin | 378 | 89 | 467 |
| Levetiracetam | 621 | 589 | 1,210 |
| Oxcarbazepine | 143 | 107 | 250 |
| Phenobarbital | 84 | 70 | 154 |
| Pregabalin | 1,627 | 150 | 1,777 |
| Topiramate | 477 | 220 | 697 |

Supplementary Table 2. Characteristics of exposure to antiepileptic drug monotherapy during pregnancy among exposed children according to the drug

|  | **Period of exposure** | | |  | **Mean daily dose**  **(mg)** |  | **Cumulative dose**  **(g)** |
| --- | --- | --- | --- | --- | --- | --- | --- |
|  | 1^st^ trimester only | 1^st^ trimester and 2^nd^ or 3^rd^ trimester | 2^nd^ or 3^rd^ trimester only |  | Mean |  | Mean |
| **Pregnancies exposed to valproate** |  |  |  |  |  |  |  |
| Valproate | 232 (23.4%) | 601 (60.6%) | 158 (15.9%) |  | 987.3 |  | 117.6 |
| **Pregnancies exposed to a drug indicated for the treatment of epilepsy and bipolar disorder** |  |  |  |  |  |  |  |
| Lamotrigine | 215 (7.6%) | 2 334 (83.0%) | 264 (9.4%) |  | 205.0 |  | 41.0 |
| Carbamazepine | 149 (31.8%) | 280 (59.8%) | 39 (8.3%) |  | 631.2 |  | 93.8 |
| **Pregnancies exposed to another antiepileptic drug** |  |  |  |  |  |  |  |
| Clonazepam | 800 (64.2%) | 233 (18.7%) | 213 (17.1%) |  | 2.1 |  | 0.1 |
| Gabapentin | 230 (60.8%) | 95 (25.1%) | 53 (14.0%) |  | 1164.6 |  | 89.4 |
| Levetiracetam | 59 (9.5%) | 470 (75.7%) | 92 (14.8%) |  | 1461.1 |  | 243.4 |
| Oxcarbazepine | 36 (25.2%) | 93 (65.0%) | 14 (9.8%) |  | 1201.1 |  | 162.1 |
| Phenobarbital | 19 (22.6%) | 51 (60.7%) | 14 (16.7%) |  | 111.4 |  | 14.2 |
| Pregabalin | 1 381 (84.9%) | 151 (9.3%) | 95 (5.8%) |  | 201.7 |  | 8.8 |
| Topiramate | 361 (75.7%) | 92 (19.3%) | 24 (5.0%) |  | 96.9 |  | 7.9 |

Supplementary Table 3. Follow-up of children in the study according to exposure to antiepileptic drug monotherapy

|  | **Follow-up**  **(years)** |  | **Death** |  | **Lost to follow-up** |
| --- | --- | --- | --- | --- | --- |
|  | Mean |  | N (%) |  | N (%) |
| **Children not exposed to antiepileptic drugs** | 3.6 |  | 4 695 (0.3%) |  | 273 489 (16.0%) |
| **Children exposed to valproate** |  |  |  |  |  |
| Valproate | 3.8 |  | 8 (0.8%) |  | 148 (14.9%) |
| **Children exposed to drugs indicated for epilepsy and bipolar disorder** |  |  |  |  |  |
| Lamotrigine | 3.6 |  | 9 (0.3%) |  | 366 (13.0%) |
| Carbamazepine | 3.7 |  | 1 (0.2%) |  | 73 (15.6%) |
| **Children exposed to another antiepileptic drug** |  |  |  |  |  |
| Clonazepam | 4.4 |  | 2 (0.2%) |  | 201 (16.1%) |
| Gabapentin | 3.4 |  | 1 (0.3%) |  | 55 (14.6%) |
| Levetiracetam | 3.5 |  | 3 (0.5%) |  | 75 (12.1%) |
| Oxcarbazepine | 3.7 |  | 1 (0.7%) |  | 17 (11.9%) |
| Phenobarbital | 3.9 |  | 0 |  | 7 (8.3%) |
| Pregabalin | 3.4 |  | 4 (0.2%) |  | 194 (11.9%) |
| Topiramate | 3.6 |  | 1 (0.2%) |  | 65 (13.6%) |

Supplementary Table 4. Diagnoses of mental and behavioural disorders: Number of cases, identification source and mean age at diagnosis

|  | Diagnosis | | LTD exclusively | |  | Hospital exclusively | |  | LTD and Hospital | |  | Total | |
| --- | --- | --- | --- | --- | --- | --- | --- | --- | --- | --- | --- | --- | --- |
|  |  |  | N (%) | Mean age (years) |  | N (%) | Mean age (years) |  | N (%) | Mean age (years) |  | N (%) | Mean age (years) |
| **Children not exposed to antiepileptic drugs** | Mental and behavioural disorders (F70-F98) | | 5727 (37.5) | 3.0 |  | 9537 (62.5) | 2.0 |  | 6 (0.0) | 2.9 |  | 15270 (100.0) | 2.4 |
|  | Pervasive developmental disorders (F84) | | 2834 (66.2) | 3.3 |  | 1444 (33.7) | 3.2 |  | 2 (0.0) | 3.5 |  | 4280 (100.0) | 3.3 |
|  | Mental retardation (F70-F79) | | 1342 (39.5) | 2.3 |  | 2055 (60.5) | 2.3 |  | 1 (0.0) | 1.5 |  | 3398 (100.0) | 2.3 |
|  | Disorders of psychological development (F80-F89) | | 4464 (44.6) | 3.2 |  | 5543 (55.4) | 2.4 |  | 3 (0.0) | 3.2 |  | 10010 (100.0) | 2.8 |
|  | Behavioural and emotional disorders with onset usually occurring in childhood and adolescence (F90-F98) | | 551 (12.5) | 3.3 |  | 3847 (87.5) | 1.7 |  | 0 (0.0) |  |  | 4398 (100.0) | 1.9 |
| **Children exposed to valproate** | | |  |  |  |  |  |  |  |  |  |  |  |
| Valproate | Mental and behavioural disorders (F70-F98) | | 19 (38.0) | 3.0 |  | 31 (62.0) | 2.2 |  | 0 (0.0) |  |  | 50 (100.0) | 2.5 |
|  | Pervasive developmental disorders (F84) | | 14 (82.4) | 3.2 |  | 3 (17.6) | 2.8 |  | 0 (0.0) |  |  | 17 (100.0) | 3.2 |
|  | Mental retardation (F70-F79) | | 5 (33.3) | 3.0 |  | 10 (66.7) | 2.6 |  | 0 (0.0) |  |  | 15 (100.0) | 2.7 |
|  | Disorders of psychological development (F80-F89) | | 17 (41.5) | 2.9 |  | 24 (58.5) | 2.6 |  | 0 (0.0) |  |  | 41 (100.0) | 2.7 |
|  | Behavioural and emotional disorders with onset usually occurring in childhood and adolescence (F90-F98) | | 0 (0.0) |  |  | 7 (100.0) | 1.6 |  | 0 (0.0) |  |  | 7 (100.0) | 1.6 |
| **Children exposed to drugs indicated for epilepsy and bipolar disorder** | | | | |  |  |  |  |  |  |  |  |  |
| Lamotrigine | | Mental and behavioural disorders (F70-F98) | 18 (38.3) | 2.7 |  | 29 (61.7) | 1.8 |  | 0 (0.0) |  |  | 47 (100.0) | 2.2 |
|  |  | Pervasive developmental disorders (F84) | 9 (90.0) | 3.1 |  | 1 (10.0) | 2.5 |  | 0 (0.0) |  |  | 10 (100.0) | 3.1 |
|  |  | Mental retardation (F70-F79) | 6 (40.0) | 2.1 |  | 9 (60.0) | 1.9 |  | 0 (0.0) |  |  | 15 (100.0) | 2.0 |
|  |  | Disorders of psychological development (F80-F89) | 14 (51.9) | 2.9 |  | 13 (48.1) | 2.1 |  | 0 (0.0) |  |  | 27 (100.0) | 2.5 |
|  |  | Behavioural and emotional disorders with onset usually occurring in childhood and adolescence (F90-F98) | 1 (10.0) | 2.1 |  | 9 (90.0) | 1.4 |  | 0 (0.0) |  |  | 10 (100.0) | 1.5 |
| Carbamazepine | | Mental and behavioural disorders (F70-F98) | 5 (45.5) | 2.7 |  | 6 (54.5) | 1.4 |  | 0 (0.0) |  |  | 11 (100.0) | 2.0 |
|  |  | Pervasive developmental disorders (F84) | 2 (66.7) | 2.2 |  | 1 (33.3) | 4.0 |  | 0 (0.0) |  |  | 3 (100.0) | 2.8 |
|  |  | Mental retardation (F70-F79) | 0 (0.0) |  |  | 2 (100.0) | 1.6 |  | 0 (0.0) |  |  | 2 (100.0) | 1.6 |
|  |  | Disorders of psychological development (F80-F89) | 4 (50.0) | 2.5 |  | 4 (50.0) | 2.3 |  | 0 (0.0) |  |  | 8 (100.0) | 2.4 |
|  |  | Behavioural and emotional disorders with onset usually occurring in childhood and adolescence (F90-F98) | 3 (50.0) | 2.7 |  | 3 (50.0) | 1.3 |  | 0 (0.0) |  |  | 6 (100.0) | 2.0 |
| **Children exposed to another antiepileptic drug** | | |  |  |  |  |  |  |  |  |  |  |  |
| Clonazepam | Mental and behavioural disorders (F70-F98) | | 11 (39.3) | 3.1 |  | 17 (60.7) | 2.2 |  | 0 (0.0) |  |  | 28 (100.0) | 2.5 |
|  | Pervasive developmental disorders (F84) | | 4 (50.0) | 3.5 |  | 4 (50.0) | 4.7 |  | 0 (0.0) |  |  | 8 (100.0) | 4.1 |
|  | Mental retardation (F70-F79) | | 2 (66.7) | 2.5 |  | 1 (33.3) | 1.1 |  | 0 (0.0) |  |  | 3 (100.0) | 2.1 |
|  | Disorders of psychological development (F80-F89) | | 10 (55.6) | 3.1 |  | 8 (44.4) | 3.6 |  | 0 (0.0) |  |  | 18 (100.0) | 3.3 |
|  | Behavioural and emotional disorders with onset usually occurring in childhood and adolescence (F90-F98) | | 1 (8.3) | 5.1 |  | 11 (91.7) | 1.5 |  | 0 (0.0) |  |  | 12 (100.0) | 1.8 |
| Gabapentin | Mental and behavioural disorders (F70-F98) | | 3 (75.0) | 2.2 |  | 1 (25.0) | 3.4 |  | 0 (0.0) |  |  | 4 (100.0) | 2.5 |
|  | Pervasive developmental disorders (F84) | | 1 (33.3) | 0.2 |  | 2 (66.7) | 3.0 |  | 0 (0.0) |  |  | 3 (100.0) | 2.1 |
|  | Mental retardation (F70-F79) | |  |  |  |  |  |  |  |  |  | 0 |  |
|  | Disorders of psychological development (F80-F89) | | 3 (75.0) | 2.2 |  | 1 (25.0) | 3.4 |  | 0 (0.0) |  |  | 4 (100.0) | 2.5 |
|  | Behavioural and emotional disorders with onset usually occurring in childhood and adolescence (F90-F98) | |  |  |  |  |  |  |  |  |  | 0 |  |
| Levetiracetam | Mental and behavioural disorders (F70-F98) | | 5 (62.5) | 3.9 |  | 3 (37.5) | 0.5 |  | 0 (0.0) |  |  | 8 (100.0) | 2.6 |
|  | Pervasive developmental disorders (F84) | | 4 (100.0) | 4.2 |  | 0 (0.0) |  |  | 0 (0.0) |  |  | 4 (100.0) | 4.2 |
|  | Mental retardation (F70-F79) | | 0 (0.0) |  |  | 1 (100.0) | 3.4 |  | 0 (0.0) |  |  | 1 (100.0) | 3.4 |
|  | Disorders of psychological development (F80-F89) | | 5 (100.0) | 3.9 |  | 0 (0.0) |  |  | 0 (0.0) |  |  | 5 (100.0) | 3.9 |
|  | Behavioural and emotional disorders with onset usually occurring in childhood and adolescence (F90-F98) | | 0 (0.0) |  |  | 3 (100.0) | 0.5 |  | 0 (0.0) |  |  | 3 (100.0) | 0.5 |
| Oxcarbazepine | Mental and behavioural disorders (F70-F98) | | 1 (33.3) | 2.0 |  | 2 (66.7) | 0.6 |  | 0 (0.0) |  |  | 3 (100.0) | 1.1 |
|  | Pervasive developmental disorders (F84) | | 1 (100.0) | 2.0 |  | 0 (0.0) |  |  | 0 (0.0) |  |  | 1 (100.0) | 2.0 |
|  | Mental retardation (F70-F79) | |  |  |  |  |  |  |  |  |  | 0 |  |
|  | Disorders of psychological development (F80-F89) | | 1 (100.0) | 2.0 |  | 0 (0.0) |  |  | 0 (0.0) |  |  | 1 (100.0) | 2.0 |
|  | Behavioural and emotional disorders with onset usually occurring in childhood and adolescence (F90-F98) | | 0 (0.0) |  |  | 2 (100.0) | 0.6 |  | 0 (0.0) |  |  | 2 (100.0) | 0.6 |
| Phenobarbital | | Mental and behavioural disorders (F70-F98) | 2 (100.0) | 3.7 |  | 0 (0.0) |  |  | 0 (0.0) |  |  | 2 (100.0) | 3.7 |
|  |  | Pervasive developmental disorders (F84) |  |  |  |  |  |  |  |  |  | 0 |  |
|  |  | Mental retardation (F70-F79) | 0 (0.0) |  |  | 1 (100.0) | 3.1 |  | 0 (0.0) |  |  | 1 (100.0) | 3.1 |
|  |  | Disorders of psychological development (F80-F89) | 1 (100.0) | 2.9 |  | 0 (0.0) |  |  | 0 (0.0) |  |  | 1 (100.0) | 2.9 |
|  |  | Behavioural and emotional disorders with onset usually occurring in childhood and adolescence (F90-F98) | 1 (100.0) | 4.4 |  | 0 (0.0) |  |  | 0 (0.0) |  |  | 1 (100.0) | 4.4 |
| Pregabalin | | Mental and behavioural disorders (F70-F98) | 13 (46.4) | 2.5 |  | 15 (53.6) | 1.6 |  | 0 (0.0) |  |  | 28 (100.0) | 2.0 |
|  |  | Pervasive developmental disorders (F84) | 6 (85.7) | 2.8 |  | 1 (14.3) | 2.3 |  | 0 (0.0) |  |  | 7 (100.0) | 2.7 |
|  |  | Mental retardation (F70-F79) | 4 (57.1) | 2.5 |  | 3 (42.9) | 1.6 |  | 0 (0.0) |  |  | 7 (100.0) | 2.1 |
|  |  | Disorders of psychological development (F80-F89) | 8 (50.0) | 2.4 |  | 8 (50.0) | 1.8 |  | 0 (0.0) |  |  | 16 (100.0) | 2.1 |
|  |  | Behavioural and emotional disorders with onset usually occurring in childhood and adolescence (F90-F98) | 2 (22.2) | 2.8 |  | 7 (77.8) | 1.6 |  | 0 (0.0) |  |  | 9 (100.0) | 1.8 |
| Topiramate | | Mental and behavioural disorders (F70-F98) | 1 (14.3) | 3.6 |  | 6 (85.7) | 1.7 |  | 0 (0.0) |  |  | 7 (100.0) | 2.0 |
|  |  | Pervasive developmental disorders (F84) | 0 (0.0) |  |  | 1 (100.0) | 1.7 |  | 0 (0.0) |  |  | 1 (100.0) | 1.7 |
|  |  | Mental retardation (F70-F79) | 1 (50.0) | 3.6 |  | 1 (50.0) | 0.6 |  | 0 (0.0) |  |  | 2 (100.0) | 2.1 |
|  |  | Disorders of psychological development (F80-F89) | 0 (0.0) |  |  | 3 (100.0) | 1.9 |  | 0 (0.0) |  |  | 3 (100.0) | 1.9 |
|  |  | Behavioural and emotional disorders with onset usually occurring in childhood and adolescence (F90-F98) | 0 (0.0) |  |  | 4 (100.0) | 1.7 |  | 0 (0.0) |  |  | 4 (100.0) | 1.7 |

Supplementary Table 5. Utilization of speech therapy, orthoptic and psychiatric services: number of cases and mean age at first utilization

|  |  | N | Mean age  (years) |
| --- | --- | --- | --- |
|  |  |  |  |
| **Children not exposed to antiepileptic drugs** | speech therapy | 72,012 | 3.8 |
|  | orthoptics | 203,489 | 1.9 |
|  | psychiatry | 22,365 | 2.8 |
| **Children exposed to valproate** |  |  |  |
| Valproate | speech therapy | 93 | 3.7 |
|  | orthoptics | 135 | 2.2 |
|  | psychiatry | 22 | 3.1 |
| **Children exposed to drugs indicated for epilepsy and bipolar disorder** |  |  |  |
| Lamotrigine | speech therapy | 149 | 3.8 |
|  | orthoptics | 410 | 1.8 |
|  | psychiatry | 49 | 2.6 |
| Carbamazepine | speech therapy | 29 | 3.6 |
|  | orthoptics | 68 | 1.9 |
|  | psychiatry | 11 | 2.5 |
| **Children exposed to another antiepileptic drug** |  |  |  |
| Clonazepam | speech therapy | 97 | 3.8 |
|  | orthoptics | 186 | 2.4 |
|  | psychiatry | 44 | 3.1 |
| Gabapentin | speech therapy | 11 | 3.4 |
|  | orthoptics | 42 | 1.4 |
|  | psychiatry | 6 | 3.1 |
| Levetiracetam | speech therapy | 22 | 3.6 |
|  | orthoptics | 95 | 1.6 |
|  | psychiatry | 12 | 2.7 |
| Oxcarbazepine | speech therapy | 13 | 3.4 |
|  | orthoptics | 31 | 1.9 |
|  | psychiatry | 3 | 3.1 |
| Phenobarbital | speech therapy | 7 | 3.3 |
|  | orthoptics | 10 | 1.7 |
|  | psychiatry | 2 | 2.0 |
| Pregabalin | speech therapy | 61 | 3.6 |
|  | orthoptics | 225 | 1.9 |
|  | psychiatry | 23 | 2.5 |
| Topiramate | speech therapy | 33 | 3.7 |
|  | orthoptics | 70 | 1.9 |
|  | psychiatry | 13 | 2.8 |

Supplementary Table 6. Maternal and neonatal characteristics by exposure group – Children born to a mother with no known mental illness

|  |  | Unexposed* | Exposed to valproate | Exposed to lamotrigine | Exposed to carbamazepine | Exposed to clonazepam | Exposed to gabapentin | Exposed to levetiracetam | Exposed to oxcarbazepine | Exposed to phenobarbital | Exposed to pregabalin | Exposed to topiramate |
| --- | --- | --- | --- | --- | --- | --- | --- | --- | --- | --- | --- | --- |
|  |  | (N=1,382,288) | (N=619) | (N=1,586) | (N=250) | (N=1,246) | (N=378) | (N=621) | (N=143) | (N=84) | (N=1,627) | (N=477) |
| **Maternal characteristics** |  |  |  |  |  |  |  |  |  |  |  |  |
| Age at the end of pregnancy (years) | Mean (standard deviation) | 29.7 (5.2) | 30.9 (5.7) | 29.6 (4.9) | 31.5 (5.4) | 32.1 (5.5) | 32.2 (5.5) | 29.3 (5.3) | 30.6 (5.7) | 31.4 (6.6) | 32.3 (5.7) | 30.5 (5.3) |
|  | < 25 | 222,733 (16.1) | 87 (14.1) | 245 (15.4) | 26 (10.4) | 118 (9.5) | 32 (8.5) | 119 (19.2) | 23 (16.1) | 18 (21.4) | 139 (8.5) | 66 (13.8) |
|  | [25-30[ | 465,188 (33.7) | 170 (27.5) | 552 (34.8) | 63 (25.2) | 302 (24.2) | 90 (23.8) | 212 (34.1) | 32 (22.4) | 15 (17.9) | 414 (25.4) | 144 (30.2) |
|  | [30-35[ | 446,478 (32.3) | 193 (31.2) | 523 (33.0) | 90 (36.0) | 380 (30.5) | 125 (33.1) | 181 (29.1) | 52 (36.4) | 20 (23.8) | 495 (30.4) | 153 (32.1) |
|  | ≥ 35 | 247,889 (17.9) | 169 (27.3) | 266 (16.8) | 71 (28.4) | 446 (35.8) | 131 (34.7) | 109 (17.6) | 36 (25.2) | 31 (36.9) | 579 (35.6) | 114 (23.9) |
| Complementary Universal Health Insurance scheme |  | 198,747 (14.4) | 166 (26.8) | 212 (13.4) | 48 (19.2) | 303 (24.3) | 82 (21.7) | 134 (21.6) | 36 (25.2) | 27 (32.1) | 404 (24.8) | 79 (16.6) |
| Salary reconstituted from the sum of maternity leave allowances | Missing data | 529,212 (38.3) | 315 (50.9) | 615 (38.8) | 114 (45.6) | 577 (46.3) | 162 (42.9) | 264 (42.5) | 67 (46.9) | 50 (59.5) | 701 (43.1) | 190 (39.8) |
|  | < €1000 ** | 58,644 (6.9) | 44 (14.5) | 95 (9.8) | 18 (13.2) | 58 (8.7) | 17 (7.9) | 43 (12.0) | 7 (9.2) | 6 (17.6) | 117 (12.6) | 27 (9.4) |
|  | €1000 - 1499 ** | 121,652 (14.3) | 63 (20.7) | 133 (13.7) | 21 (15.4) | 126 (18.8) | 50 (23.1) | 73 (20.4) | 18 (23.7) | 9 (26.5) | 177 (19.1) | 63 (22.0) |
|  | €1500 - 1999 ** | 280,155 (32.8) | 98 (32.2) | 356 (36.7) | 52 (38.2) | 216 (32.3) | 81 (37.5) | 118 (33.1) | 26 (34.2) | 10 (29.4) | 366 (39.5) | 103 (35.9) |
|  | €2000 - 2499 ** | 183,603 (21.5) | 57 (18.8) | 198 (20.4) | 33 (24.3) | 135 (20.2) | 34 (15.7) | 56 (15.7) | 17 (22.4) | 7 (20.6) | 136 (14.7) | 61 (21.3) |
|  | €2500 - 2999 ** | 99,424 (11.7) | 26 (8.6) | 92 (9.5) | 6 (4.4) | 48 (7.2) | 19 (8.8) | 37 (10.4) | 5 (6.6) | 1 (2.9) | 74 (8.0) | 16 (5.6) |
|  | ≥ €3000 ** | 109,598 (12.8) | 16 (5.3) | 97 (10.0) | 6 (4.4) | 86 (12.9) | 15 (6.9) | 30 (8.4) | 3 (3.9) | 1 (2.9) | 56 (6.0) | 17 (5.9) |
| Year of end of pregnancy | 2011 | 257,533 (18.6) | 145 (23.4) | 279 (17.6) | 60 (24.0) | 601 (48.2) | 59 (15.6) | 90 (14.5) | 32 (22.4) | 15 (17.9) | 230 (14.1) | 93 (19.5) |
|  | 2012 | 393,842 (28.5) | 212 (34.2) | 458 (28.9) | 73 (29.2) | 500 (40.1) | 90 (23.8) | 147 (23.7) | 50 (35.0) | 32 (38.1) | 412 (25.3) | 118 (24.7) |
|  | 2013 | 381,821 (27.6) | 148 (23.9) | 440 (27.7) | 72 (28.8) | 83 (6.7) | 116 (30.7) | 202 (32.5) | 27 (18.9) | 23 (27.4) | 465 (28.6) | 128 (26.8) |
|  | 2014 | 349,092 (25.3) | 114 (18.4) | 409 (25.8) | 45 (18.0) | 62 (5.0) | 113 (29.9) | 182 (29.3) | 34 (23.8) | 14 (16.7) | 520 (32.0) | 138 (28.9) |
| Folic acid |  | 386,886 (28.0) | 330 (53.3) | 1,142 (72.0) | 152 (60.8) | 360 (28.9) | 139 (36.8) | 396 (63.8) | 89 (62.2) | 32 (38.1) | 440 (27.0) | 179 (37.5) |
| Indicator of alcohol use | | 1,141 (0.1) | 2 (0.3) | 3 (0.2) | 0 (0.0) | 35 (2.8) | 6 (1.6) | 13 (2.1) | 1 (0.7) | 2 (2.4) | 21 (1.3) | 7 (1.5) |
| Indicator of tobacco use | | 89,592 (6.5) | 63 (10.2) | 109 (6.9) | 14 (5.6) | 165 (13.2) | 66 (17.5) | 69 (11.1) | 13 (9.1) | 8 (9.5) | 257 (15.8) | 43 (9.0) |
| Diagnosis of mental illness other than tobacco and alcohol use disorders | | 0 (0.0) | 0 (0.0) | 0 (0.0) | 0 (0.0) | 216 (17.3) | 43 (11.4) | 51 (8.2) | 36 (25.2) | 15 (17.9) | 169 (10.4) | 54 (11.3) |
| Antidepressants before pregnancy | | 0 (0.0) | 0 (0.0) | 0 (0.0) | 0 (0.0) | 494 (39.6) | 143 (37.8) | 68 (11.0) | 37 (25.9) | 13 (15.5) | 603 (37.1) | 185 (38.8) |
| Antidepressants during pregnancy | | 3,962 (0.3) | 5 (0.8) | 8 (0.5) | 4 (1.6) | 393 (31.5) | 100 (26.5) | 30 (4.8) | 29 (20.3) | 7 (8.3) | 422 (25.9) | 113 (23.7) |
| SSRI during pregnancy | | 2,346 (0.2) | 2 (0.3) | 5 (0.3) | 1 (0.4) | 201 (16.1) | 42 (11.1) | 23 (3.7) | 22 (15.4) | 5 (6.0) | 172 (10.6) | 55 (11.5) |
| Antipsychotics before pregnancy | | 0 (0.0) | 0 (0.0) | 0 (0.0) | 0 (0.0) | 123 (9.9) | 24 (6.3) | 15 (2.4) | 31 (21.7) | 6 (7.1) | 79 (4.9) | 30 (6.3) |
| Antipsychotics during pregnancy | | 4,621 (0.3) | 3 (0.5) | 8 (0.5) | 2 (0.8) | 109 (8.7) | 17 (4.5) | 8 (1.3) | 24 (16.8) | 5 (6.0) | 67 (4.1) | 27 (5.7) |
| Anxiolytics before pregnancy | | 0 (0.0) | 0 (0.0) | 0 (0.0) | 0 (0.0) | 522 (41.9) | 150 (39.7) | 232 (37.4) | 59 (41.3) | 28 (33.3) | 659 (40.5) | 188 (39.4) |
| Anxiolytics during pregnancy | | 44,092 (3.2) | 60 (9.7) | 193 (12.2) | 23 (9.2) | 389 (31.2) | 112 (29.6) | 173 (27.9) | 50 (35.0) | 23 (27.4) | 429 (26.4) | 103 (21.6) |
| Hypnotics before pregnancy | | 0 (0.0) | 0 (0.0) | 0 (0.0) | 0 (0.0) | 287 (23.0) | 72 (19.0) | 57 (9.2) | 20 (14.0) | 12 (14.3) | 315 (19.4) | 71 (14.9) |
| Hypnotics during pregnancy | | 3,427 (0.2) | 1 (0.2) | 3 (0.2) | 4 (1.6) | 157 (12.6) | 32 (8.5) | 28 (4.5) | 15 (10.5) | 4 (4.8) | 149 (9.2) | 34 (7.1) |
| Indicator of severity of psychiatric morbidity (number of ATC classes of psychotropic drugs) | 0 | 1,382,288 (100.0) | 619 (100.0) | 1,586 (100.0) | 250 (100.0) | 492 (39.5) | 157 (41.5) | 366 (58.9) | 65 (45.5) | 53 (63.1) | 701 (43.1) | 217 (45.5) |
|  | 1 | 0 (0.0) | 0 (0.0) | 0 (0.0) | 0 (0.0) | 283 (22.7) | 91 (24.1) | 138 (22.2) | 33 (23.1) | 10 (11.9) | 355 (21.8) | 92 (19.3) |
|  | 2 | 0 (0.0) | 0 (0.0) | 0 (0.0) | 0 (0.0) | 149 (12.0) | 49 (13.0) | 54 (8.7) | 14 (9.8) | 8 (9.5) | 227 (14.0) | 69 (14.5) |
|  | 3 | 0 (0.0) | 0 (0.0) | 0 (0.0) | 0 (0.0) | 102 (8.2) | 40 (10.6) | 28 (4.5) | 5 (3.5) | 4 (4.8) | 133 (8.2) | 36 (7.5) |
|  | 4 | 0 (0.0) | 0 (0.0) | 0 (0.0) | 0 (0.0) | 81 (6.5) | 14 (3.7) | 11 (1.8) | 4 (2.8) | 5 (6.0) | 83 (5.1) | 32 (6.7) |
|  | ≥5 | 0 (0.0) | 0 (0.0) | 0 (0.0) | 0 (0.0) | 139 (11.2) | 27 (7.2) | 24 (3.9) | 22 (15.4) | 4 (4.8) | 128 (7.9) | 31 (6.5) |
| **Neonatal characteristics** |  |  |  |  |  |  |  |  |  |  |  |  |
| Gestational age at birth | Mean (standard deviation) | 39.2 (1.7) | 39.2 (1.7) | 39.1 (1.6) | 39.1 (1.5) | 38.9 (1.9) | 38.6 (2.1) | 38.9 (2.0) | 38.8 (1.9) | 38.8 (2.0) | 38.9 (1.9) | 39.0 (2.1) |
|  | 22 - 26 WA | 1,572 (0.1) | 0 (0.0) | 2 (0.1) | 0 (0.0) | 1 (0.1) | 0 (0.0) | 1 (0.2) | 1 (0.7) | 0 (0.0) | 3 (0.2) | 1 (0.2) |
|  | 27 - 31 WA | 6,630 (0.5) | 3 (0.5) | 3 (0.2) | 1 (0.4) | 9 (0.7) | 2 (0.5) | 6 (1.0) | 0 (0.0) | 1 (1.2) | 9 (0.6) | 6 (1.3) |
|  | 32 - 34 WA | 15,969 (1.2) | 11 (1.8) | 22 (1.4) | 3 (1.2) | 28 (2.2) | 23 (6.1) | 11 (1.8) | 3 (2.1) | 1 (1.2) | 37 (2.3) | 10 (2.1) |
|  | 35 - 36 WA | 46,456 (3.4) | 28 (4.5) | 67 (4.2) | 4 (1.6) | 55 (4.4) | 21 (5.6) | 33 (5.3) | 6 (4.2) | 3 (3.6) | 61 (3.7) | 19 (4.0) |
|  | ≥ 37 WA | 1,311,661 (94.9) | 577 (93.2) | 1,492 (94.1) | 242 (96.8) | 1153 (92.5) | 332 (87.8) | 570 (91.8) | 133 (93.0) | 79 (94.0) | 1,517 (93.2) | 441 (92.5) |
| Sex | Male | 706,772 (51.1) | 310 (50.1) | 840 (53.0) | 125 (50.0) | 597 (47.9) | 206 (54.5) | 292 (47.0) | 65 (45.5) | 43 (51.2) | 847 (52.1) | 254 (53.2) |
| Weight at birth | Mean (standard deviation) | 3,297 (509) | 3,230 (537) | 3,284 (512) | 3,289 (501) | 3,220 (553) | 3,190 (600) | 3,152 (540) | 3,218 (597) | 3,087 (593) | 3,263 (563) | 3,257 (579) |
|  | < 2500 g | 70,062 (5.1) | 51 (8.2) | 86 (5.4) | 16 (6.4) | 105 (8.4) | 47 (12.4) | 60 (9.7) | 12 (8.4) | 12 (14.3) | 118 (7.3) | 32 (6.7) |
|  | 2500 - 2999 g | 267,164 (19.3) | 140 (22.6) | 334 (21.1) | 54 (21.6) | 278 (22.3) | 73 (19.3) | 152 (24.5) | 30 (21.0) | 20 (23.8) | 324 (19.9) | 90 (18.9) |
|  | 3000 - 3499 g | 572,274 (41.4) | 246 (39.7) | 654 (41.2) | 89 (35.6) | 485 (38.9) | 140 (37.0) | 251 (40.4) | 60 (42.0) | 35 (41.7) | 630 (38.7) | 203 (42.6) |
|  | ≥ 3500 g | 472,788 (34.2) | 182 (29.4) | 512 (32.3) | 91 (36.4) | 378 (30.3) | 118 (31.2) | 158 (25.4) | 41 (28.7) | 17 (20.2) | 555 (34.1) | 152 (31.9) |
| Small for gestational age | | 131,180 (9.5) | 90 (14.5) | 152 (9.6) | 26 (10.4) | 159 (12.8) | 45 (11.9) | 82 (13.2) | 14 (9.8) | 17 (20.2) | 177 (10.9) | 55 (11.5) |
| Neonatal condition | | 179,477 (13.0) | 66 (10.7) | 187 (11.8) | 30 (12.0) | 197 (15.8) | 53 (14.0) | 85 (13.7) | 17 (11.9) | 9 (10.7) | 231 (14.2) | 74 (15.5) |

^*^ Not exposed to any antiepileptic drug during pregnancy ^**^ among those for whom the information was available

Supplementary Table 7. Incidence of early neurodevelopmental disorders during follow-up according to exposure to valproate during pregnancy, compared to children exposed to lamotrigine during pregnancy, in the overall study population

|  | Children exposed to valproate during pregnancy  (N=991) | |  | Children exposed to lamotrigine  during pregnancy  (N=2,916) | | aHR [95%CI]* |
| --- | --- | --- | --- | --- | --- | --- |
|  | N | IR  per 1,000 PY |  | N | IR  per 1,000 PY |  |
| **Diagnosis** |  |  |  |  |  |  |
| Mental and behavioural disorders (F70-F98) | 50 | 13.5 |  | 51 | 4.9 | **2.6 [1.7 - 3.9]** |
| Pervasive developmental disorders (F84) | 17 | 4.5 |  | 11 | 1.1 | **4.0 [1.8 - 8.9]** |
| Mental retardation (F70-F79) | 15 | 4.0 |  | 15 | 1.4 | **2.9 [1.4 - 6.1]** |
| Disorders of psychological development (F80-F89) | 41 | 11.0 |  | 30 | 2.9 | **3.5 [2.1 - 5.7]** |
| Behavioural and emotional disorders with onset usually occurring in childhood and adolescence (F90-F98) | 7 | 1.8 |  | 11 | 1.1 | 1.7 [0.6 - 4.6] |
| **Health care utilization** |  |  |  |  |  |  |
| Speech therapy | 93 | 25.1 |  | 157 | 15.2 | **1.5 [1.2 - 2.0]** |
| Orthoptics | 135 | 38.4 |  | 425 | 44.2 | 1.0 [0.8 - 1.2] |
| Psychiatry | 22 | 5.8 |  | 60 | 5.8 | 1.1 [0.7 - 1.9] |

*IR: Incidence rate IRR: Incidence rate ratio HR: Hazard ratio 95%CI: 95% confidence interval*

** Cox models adjusted for: mother’s age, Complementary Universal Health Insurance scheme, diagnosis of mental illness other than tobacco and alcohol use disorders, antipsychotics during the year preceding pregnancy, indicator of severity of psychiatric morbidity, indicator of tobacco use, indicator of alcohol use, folic acid, SSRI during pregnancy, child’s sex, gestational age and birth weight, except:*

*- F70-F98: gestational age according to 3 classes (< 35 WA, 35-36 WA, ≥ 37 WA)*

*- F84: gestational age according to 3 classes (< 35 WA, 35-36 WA, ≥ 37 WA), indicator of imbalance according to 4 classes (0, 1-2, 3-4, 5 or more), no adjustment for alcohol and SSRI during pregnancy*

*- F70-F79: no adjustment for gestational age, Complementary Universal Health Insurance scheme, alcohol and SSRI during pregnancy, indicator of imbalance according to 4 classes (0, 1-2, 3-4, 5 or more), and mother’s age according to 3 classes (< 30 years, 30-34 years, ≥ 35 years)*

*- F80-F89: gestational age according to 3 classes (< 35 WA, 35-36 WA, ≥ 37 WA),*

*- F90-F98: gestational age according to 2 classes (< 37 WA, ≥ 37 WA), birth weight according to 3 classes (< 3000 g, 3000-3499 g, ≥ 3500 g)*

*- speech therapy/orthoptics: gestational age according to 4 classes (< 32 WA, 32-34 WA, 35-36 WA, ≥ 37 WA),*

*- psychiatry: gestational age according to 3 classes (< 35 WA, 35-36 WA, ≥ 37 WA)*

Supplementary Table 8. Incidence of early neurodevelopmental disorders during follow-up according to the mean daily exposure dose to VPA during pregnancy in the overall study population (compared to unexposed children)

|  | Unexposed children  (N=1,710,441) | |  | Children exposed to valproate  1^st^ tertile of mean daily dose (≤730 mg/day)  (N=328) | | | |  | | Children exposed to valproate  2^nd^ tertile of mean daily dose (731 to 1100 mg/day)  (N=327) | | | | | |  | | Children exposed to valproate  3^rd^ tertile of mean daily dose (>1100 mg/day)  (N=336) | | | | |
| --- | --- | --- | --- | --- | --- | --- | --- | --- | --- | --- | --- | --- | --- | --- | --- | --- | --- | --- | --- | --- | --- | --- |
|  | N | IR per 1,000 PY |  | N | IR per 1,000 PY | aHR [95%CI]* | |  | | N | | IR per 1,000 PY | | aHR [95%CI]* | |  | | N | | IR per 1,000 PY | | aHR [95%CI]* |
| **Diagnosis** |  |  |  |  |  |  |  | |  | |  | |  | |  | |  | |  | |  | |
| Mental and behavioural disorders (F70-F98) | 15,270 | 2.5 |  | 8 | 6.7 | **2.0 [1.0 - 4.0]^a^** | |  | | 10 | | 7.8 | | **2.2 [1.2 - 4.2]** | |  | | 32 | | 25.8 | | **6.5 [4.6 - 9.2]** |
| Pervasive developmental disorders (F84) | 4,280 | 0.7 |  | 2 | 1.7 | 1.8 [0.5 - 7.2] | |  | | 3 | | 2.3 | | 2.5 [0.8 - 7.6] | |  | | 12 | | 9.3 | | **9.0 [5.1 - 16.0]** |
| Mental retardation (F70-F79) | 3,398 | 0.6 |  | 5 | 4.1 | **5.9 [2.5 - 14.2]** | |  | | 2 | | 1.5 | | 2.1 [0.5 - 8.4] | |  | | 8 | | 6.2 | | **7.1 [3.5 - 14.2]** |
| Disorders of psychological development (F80-F89) | 10,010 | 1.6 |  | 6 | 5.0 | **2.3 [1.0 - 5.1]^b^** | |  | | 8 | | 6.3 | | **2.8 [1.4 - 5.5]** | |  | | 27 | | 21.4 | | **8.4 [5.8 - 12.3]** |
| Behavioural and emotional disorders with onset usually occurring in childhood and adolescence (F90-F98) | 4,398 | 0.7 |  | 1 | 0.8 | 0.8 [0.1 - 5.6] | |  | | 2 | | 1.5 | | 1.5 [0.4 - 5.8] | |  | | 4 | | 3.1 | | 2.6 [1.0 - 6.8]^d^ |
| **Health care utilization** |  |  |  |  |  |  |  | |  | |  | |  | |  | |  | |  | |  | |
| Speech therapy | 72,012 | 11.9 |  | 14 | 11.6 | 0.8 [0.5 - 1.4] | |  | | 28 | | 22.3 | | **1.4 [1.0 - 2.1]^c^** | |  | | 51 | | 41.3 | | **2.9 [2.2 - 3.8]** |
| Orthoptics | 203,489 | 35.6 |  | 39 | 34.0 | 1.0 [0.7 - 1.3] | |  | | 44 | | 36.8 | | 1.0 [0.7 - 1.3] | |  | | 52 | | 44.4 | | 1.2 [0.9 - 1.6] |
| Psychiatry | 22,365 | 3.7 |  | 7 | 5.8 | 1.3 [0.6 - 2.8] | |  | | 6 | | 4.7 | | 1.0 [0.4 - 2.2] | |  | | 9 | | 7.0 | | 1.4 [0.8 - 2.8] |

*IR: Incidence rate HR: Hazard ratio 95%CI: 95% confidence interval*

** Cox models adjusted for: mother’s age, Complementary Universal Health Insurance scheme, diagnosis of mental illness other than tobacco and alcohol use disorders, antipsychotics during the year preceding pregnancy, indicator of severity of psychiatric morbidity, indicator of tobacco use, indicator of alcohol use, folic acid, SSRI during pregnancy, child’s sex, gestational age and birth weight*

*^a^ p=0.05*

*^b^ p=0.04*

*^c^ p=0.05*

*^d^ p=0.06*

Supplementary Table 9. Incidence of early neurodevelopmental disorders during follow-up according to exposure to carbamazepine during pregnancy (compared to children not exposed to an antiepileptic drug during pregnancy) in the overall study population and among children born to a mother with no known mental illness

|  | **Overall population** | | | | | |  | **Children born to a mother with no known mental illness** | | | | | |
| --- | --- | --- | --- | --- | --- | --- | --- | --- | --- | --- | --- | --- | --- |
|  | Children exposed to carbamazepine during pregnancy  (N=468) | |  | Unexposed children  (N=1,707,707) | | aHR [95%CI]* |  | Children exposed to carbamazepine during pregnancy  (N=250) | |  | Unexposed children  (N=1,382,176) | | aHR [95%CI]** |
|  | N | IR  per 1,000 PY |  | N | IR  per 1,000 PY |  |  | N | IR  per 1,000 PY |  | N | IR  per 1,000 PY |  |
| **Diagnosis** |  |  |  |  |  |  |  |  |  |  |  |  |  |
| Mental and behavioural disorders (F70-F98) | 11 | 6.3 |  | 15,165 | 2.5 | **1.9 [1.0 - 3.4]^a^** |  | 2 | 2.2 |  | 11,010 | 2.2 | 0.9 [0.2 - 3.7] |
| Pervasive developmental disorders (F84) | 3 | 1.7 |  | 4,254 | 0.7 | 1.7 [0.5 - 5.2] |  | 0 | 0.0 |  | 3,131 | 0.6 | - |
| Mental retardation (F70-F79) | 2 | 1.1 |  | 3,385 | 0.6 | 1.6 [0.4 - 6.6] |  | 1 | 1.1 |  | 2,544 | 0.5 | 2.0 [0.3 - 14.2] |
| Disorders of psychological development (F80-F89) | 8 | 4.6 |  | 9,949 | 1.6 | **2.0 [1.0 - 4.1]^b^** |  | 1 | 1.1 |  | 7,315 | 1.5 | 0.7 [0.1 - 4.8] |
| Behavioural and emotional disorders with onset usually occurring in childhood and adolescence (F90-F98) | 6 | 3.4 |  | 4,352 | 0.7 | **3.2 [1.4 - 7.1]** |  | 0 | 0.0 |  | 2,974 | 0.6 | - |
| **Health care utilization** |  |  |  |  |  |  |  |  |  |  |  |  |  |
| Speech therapy | 29 | 16.7 |  | 71,832 | 11.8 | 1.2 [0.8 - 1.7] |  | 12 | 13.0 |  | 55,527 | 11.3 | 1.1 [0.6 - 1.9] |
| Orthoptics | 68 | 42.0 |  | 203,120 | 35.6 | 1.1 [0.9 - 1.4] |  | 38 | 45.4 |  | 162,414 | 35.3 | 1.3 [0.9 - 1.7] |
| Psychiatry | 11 | 6.3 |  | 22,261 | 3.6 | 1.1 [0.6 - 2.0] |  | 4 | 4.3 |  | 16,015 | 3.3 | 1.2 [0.4 - 3.2] |

*IR: Incidence rate IRR: Incidence rate ratio HR: Hazard ratio 95%CI: 95% confidence interval*

** Cox models adjusted for: mother’s age, Complementary Universal Health Insurance scheme, diagnosis of mental illness other than tobacco and alcohol use disorders, antipsychotics during the year preceding pregnancy, indicator of severity of psychiatric morbidity, indicator of tobacco use, indicator of alcohol use, folic acid, SSRI during pregnancy, child’s sex, gestational age and birth weight*

*** Cox models adjusted for: mother’s age, Complementary Universal Health Insurance scheme, indicator of tobacco use, indicator of alcohol use, folic acid, child’s sex, gestational age and birth weight*

*^a^ p=0.04 ^b^ p=0.05*

Supplementary Table 10. Incidence of early neurodevelopmental disorders during follow-up according to exposure to clonazepam during pregnancy, compared to unexposed children

|  | **Overall population** | | | | | |  | **Children born to a mother with no known mental illness** |
| --- | --- | --- | --- | --- | --- | --- | --- | --- |
|  | Children exposed to clonazepam during pregnancy  (N=1,246) | |  | Unexposed children  (N=1,710,441) | | aHR [95%CI]* |  | aHR [95%CI]** |
|  | N | IR  per 1,000 PY |  | N | IR  per 1,000 PY |  |  |  |
| **Diagnosis** |  |  |  |  |  |  |  |  |
| Mental and behavioural disorders (F70-F98) | 28 | 5.1 |  | 15,270 | 2.5 | 1.1 [0.8 - 1.6] |  | 0.9 [0.4 - 2.2] |
| Pervasive developmental disorders (F84) | 8 | 1.4 |  | 4,280 | 0.7 | 1.1 [0.5 - 2.1] |  | 1.1 [0.3 - 4.5] |
| Mental retardation (F70-F79) | 3 | 0.5 |  | 3,398 | 0.6 | 0.6 [0.2 - 1.9] |  | 1.7 [0.4 - 6.6] |
| Disorders of psychological development (F80-F89) | 18 | 3.2 |  | 10,010 | 1.6 | 1.1 [0.7 - 1.7] |  | 1.3 [0.5 - 3.1] |
| Behavioural and emotional disorders with onset usually occurring in childhood and adolescence (F90-F98) | 12 | 2.2 |  | 4,398 | 0.7 | 1.4 [0.8 - 2.5] |  | - |
| **Health care utilization** |  |  |  |  |  |  |  |  |
| Speech therapy | 97 | 17.7 |  | 72,012 | 11.9 | 0.9 [0.7 - 1.1] |  | 0.8 [0.6 - 1.2] |
| Orthoptics | 186 | 36.1 |  | 203,489 | 35.6 | 1.0 [0.8 - 1.1] |  | 0.9 [0.7 - 1.2] |
| Psychiatry | 44 | 8.0 |  | 22,365 | 3.7 | 1.2 [0.9 - 1.7] |  | 1.2 [0.7 - 2.3] |

*IR: Incidence rate IRR: Incidence rate ratio HR: Hazard ratio 95%CI: 95% confidence interval*

** Cox models adjusted for: mother’s age, Complementary Universal Health Insurance scheme, diagnosis of mental illness other than tobacco and alcohol use disorders, antipsychotics during the year preceding pregnancy, indicator of severity of psychiatric morbidity, indicator of tobacco use, indicator of alcohol use, folic acid, SSRI during pregnancy, child’s sex, gestational age and birth weight*

*** Cox models adjusted for: mother’s age, Complementary Universal Health Insurance scheme, indicator of tobacco use, indicator of alcohol use, folic acid, child’s sex, gestational age and birth weight*

Supplementary Table 11. Incidence of early neurodevelopmental disorders during follow-up according to exposure to gabapentin during pregnancy, compared to unexposed children

|  | **Overall population** | | | | | |  | **Children born to a mother with no known mental illness** |
| --- | --- | --- | --- | --- | --- | --- | --- | --- |
|  | Children exposed to gabapentin during pregnancy  (N=378) | |  | Unexposed children  (N=1,710,441) | | aHR [95%CI]* |  | aHR [95%CI]** |
|  | N | IR  per 1,000 PY |  | N | IR  per 1,000 PY |  |  |  |
| **Diagnosis** |  |  |  |  |  |  |  |  |
| Mental and behavioural disorders (F70-F98) | 4 | 3.1 |  | 15,270 | 2.5 | 0.8 [0.3 - 2.1] |  | 1.4 [0.4 - 5.7] |
| Pervasive developmental disorders (F84) | 3 | 2.3 |  | 4,280 | 0.7 | 2.3 [0.8 - 7.3] |  | 2.7 [0.4 - 19.2] |
| Mental retardation (F70-F79) | 0 | 0.0 |  | 3,398 | 0.6 | - |  | - |
| Disorders of psychological development (F80-F89) | 4 | 3.1 |  | 10,010 | 1.6 | 1.3 [0.5 - 3.3] |  | 2.2 [0.5 - 8.6] |
| Behavioural and emotional disorders with onset usually occurring in childhood and adolescence (F90-F98) | 0 | 0.0 |  | 4,398 | 0.7 | - |  | - |
| **Health care utilization** |  |  |  |  |  |  |  |  |
| Speech therapy | 11 | 8.5 |  | 72,012 | 11.9 | 0.6 [0.4 - 1.2] |  | 0.9 [0.4 - 2.1] |
| Orthoptics | 42 | 35.1 |  | 203,489 | 35.6 | 0.9 [0.7 - 1.2] |  | 1.1 [0.7 - 1.7] |
| Psychiatry | 6 | 4.6 |  | 22,365 | 3.7 | 0.9 [0.4 - 2.0] |  | 1.7 [0.5 - 5.3] |

*IR: Incidence rate IRR: Incidence rate ratio HR: Hazard ratio 95%CI: 95% confidence interval*

** Cox models adjusted for: mother’s age, Complementary Universal Health Insurance scheme, diagnosis of mental illness other than tobacco and alcohol use disorders, antipsychotics during the year preceding pregnancy, indicator of severity of psychiatric morbidity, indicator of tobacco use, indicator of alcohol use, folic acid, SSRI during pregnancy, child’s sex, gestational age and birth weight*

*** Cox models adjusted for: mother’s age, Complementary Universal Health Insurance scheme, indicator of tobacco use, indicator of alcohol use, folic acid, child’s sex, gestational age and birth weight*

Supplementary Table 12. Incidence of early neurodevelopmental disorders during follow-up according to exposure to levetiracetam during pregnancy, compared to unexposed children

|  | **Overall population** | | | | | |  | | **Children born to a mother with no known mental illness** | |  |
| --- | --- | --- | --- | --- | --- | --- | --- | --- | --- | --- | --- |
|  | Children exposed to levetiracetam during pregnancy  (N=621) | |  | Unexposed children  (N=1,710,441) | | aHR [95%CI]* | |  | | aHR [95%CI]** | |
|  | N | IR  per 1,000 PY |  | N | IR  per 1,000 PY |  |  |  |  |  |  |
| **Diagnosis** |  |  |  |  |  |  | |  | |  | |
| Mental and behavioural disorders (F70-F98) | 8 | 3.7 |  | 15,270 | 2.5 | 1.2 [0.6 - 2.4] | |  | | 1.5 [0.6 - 4.1] | |
| Pervasive developmental disorders (F84) | 4 | 1.8 |  | 4,280 | 0.7 | 2.5 [0.9 - 6.6] | |  | | 3.0 [0.8 - 12.2] | |
| Mental retardation (F70-F79) | 1 | 0.5 |  | 3,398 | 0.6 | 0.7 [0.1 - 4.9] | |  | | - | |
| Disorders of psychological development (F80-F89) | 5 | 2.3 |  | 10,010 | 1.6 | 1.2 [0.5 - 2.9] | |  | | 1.2 [0.3 - 4.8] | |
| Behavioural and emotional disorders with onset usually occurring in childhood and adolescence (F90-F98) | 3 | 1.4 |  | 4,398 | 0.7 | 1.3 [0.4 - 4.1] | |  | | 2.6 [0.7 - 10.4] | |
| **Health care utilization** |  |  |  |  |  |  | |  | |  | |
| Speech therapy | 22 | 10.2 |  | 72,012 | 11.9 | 0.8 [0.6 - 1.3] | |  | | 1.2 [0.7 - 2.0] | |
| Orthoptics | 95 | 48.4 |  | 203,489 | 35.6 | **1.2 [1.0 - 1.5]^a^** | |  | | **1.3 [1.0 - 1.7]^b^** | |
| Psychiatry | 12 | 5.6 |  | 22,365 | 3.7 | 1.2 [0.7 - 2.2] | |  | | **2.0 [1.0 - 4.1]^c^** | |

*IR: Incidence rate IRR: Incidence rate ratio HR: Hazard ratio 95%CI: 95% confidence interval*

** Cox models adjusted for: mother’s age, Complementary Universal Health Insurance scheme, diagnosis of mental illness other than tobacco and alcohol use disorders, antipsychotics during the year preceding pregnancy, indicator of severity of psychiatric morbidity, indicator of tobacco use, indicator of alcohol use, folic acid, SSRI during pregnancy, child’s sex, gestational age and birth weight*

*** Cox models adjusted for: mother’s age, Complementary Universal Health Insurance scheme, indicator of tobacco use, indicator of alcohol use, folic acid, child’s sex, gestational age and birth weight*

*^a^ p=0.03*

*^b^ p=0.04*

*^c^ p=0.04*

Supplementary Table 13. Incidence of early neurodevelopmental disorders during follow-up according to exposure to oxcarbazepine during pregnancy (compared to unexposed children)

|  | **Overall population** | | | | | |  | **Children born to a mother with no known mental illness** |
| --- | --- | --- | --- | --- | --- | --- | --- | --- |
|  | Enfants exposed to oxcarbazepine during pregnancy  (N=143) | |  | Unexposed children  (N=1,710,441) | | aHR [95%CI]* |  | aHR [95%CI]** |
|  | N | IR  per 1,000 PY |  | N | IR  per 1,000 PY |  |  |  |
| **Diagnosis** |  |  |  |  |  |  |  |  |
| Mental and behavioural disorders (F70-F98) | 3 | 5.6 |  | 15,270 | 2.5 | 1.4 [0.5 - 4.4] |  | - |
| Pervasive developmental disorders (F84) | 1 | 1.9 |  | 4,280 | 0.7 | 1.7 [0.2 - 12.4] |  | - |
| Mental retardation (F70-F79) | 0 | 0.0 |  | 3,398 | 0.6 | - |  | - |
| Disorders of psychological development (F80-F89) | 1 | 1.9 |  | 10,010 | 1.6 | 0.7 [0.1 - 5.3] |  | - |
| Behavioural and emotional disorders with onset usually occurring in childhood and adolescence (F90-F98) | 2 | 3.7 |  | 4,398 | 0.7 | 2.7 [0.7 - 10.7] |  | - |
| **Health care utilization** |  |  |  |  |  |  |  |  |
| Speech therapy | 13 | 24.6 |  | 72,012 | 11.9 | 1.7 [1.0 - 2.9]^a^ |  | 1.3 [0.5 - 3.5] |
| Orthoptics | 31 | 64.7 |  | 203,489 | 35.6 | **1.7 [1.2 - 2.4]** |  | 1.2 [0.6 - 2.3] |
| Psychiatry | 3 | 5.6 |  | 22,365 | 3.7 | 0.9 [0.3 - 2.7] |  | 2.5 [0.6 - 10.0] |

*IR: Incidence rate IRR: Incidence rate ratio HR: Hazard ratio 95%CI: 95% confidence interval*

** Cox models adjusted for: mother’s age, Complementary Universal Health Insurance scheme, diagnosis of mental illness other than tobacco and alcohol use disorders, antipsychotics during the year preceding pregnancy, indicator of severity of psychiatric morbidity, indicator of tobacco use, indicator of alcohol use, folic acid, SSRI during pregnancy, child’s sex, gestational age and birth weight*

*** Cox models adjusted for: mother’s age, Complementary Universal Health Insurance scheme, indicator of tobacco use, indicator of alcohol use, folic acid, child’s sex, gestational age and birth weight*

*^a^ p=0.06*

Supplementary Table 14. Incidence of early neurodevelopmental disorders during follow-up according to exposure to phenobarbital during pregnancy, compared to unexposed children

|  | **Overall population** | | | | | |  | **Children born to a mother with no known mental illness** |
| --- | --- | --- | --- | --- | --- | --- | --- | --- |
|  | Children exposed to phenobarbital during pregnancy  (N=84) | |  | Unexposed children  (N=1,710,441) | | aHR [95%CI]* |  | aHR [95%CI]** |
|  | N | IR  per 1,000 PY |  | N | IR  per 1,000 PY |  |  |  |
| **Diagnosis** |  |  |  |  |  |  |  |  |
| Mental and behavioural disorders (F70-F98) | 2 | 6.1 |  | 15,270 | 2.5 | 1.2 [0.3 - 5.0] |  | 2.0 [0.3 - 14.0] |
| Pervasive developmental disorders (F84) | 0 | 0.0 |  | 4,280 | 0.7 | - |  | - |
| Mental retardation (F70-F79) | 1 | 3.0 |  | 3,398 | 0.6 | 3.0 [0.4 - 21.4] |  | - |
| Disorders of psychological development (F80-F89) | 1 | 3.0 |  | 10,010 | 1.6 | 0.9 [0.1 - 6.7] |  | - |
| Behavioural and emotional disorders with onset usually occurring in childhood and adolescence (F90-F98) | 1 | 3.0 |  | 4,398 | 0.7 | 2.1 [0.3 - 14.9] |  | **7.6 [1.1 - 53.6]** |
| **Health care utilization** |  |  |  |  |  |  |  |  |
| Speech therapy | 7 | 21.7 |  | 72,012 | 11.9 | 1.5 [0.7 - 3.2] |  | 1.4 [0.4 - 4.2] |
| Orthoptics | 10 | 32.8 |  | 203,489 | 35.6 | 0.9 [0.5 - 1.7] |  | 1.0 [0.4 - 2.2] |
| Psychiatry | 2 | 6.1 |  | 22,365 | 3.7 | 1.2 [0.3 - 4.7] |  | 3.1 [0.8 - 12.2] |

*IR: Incidence rate IRR: Incidence rate ratio HR: Hazard ratio 95%CI: 95% confidence interval*

** Cox models adjusted for: mother’s age, Complementary Universal Health Insurance scheme, diagnosis of mental illness other than tobacco and alcohol use disorders, antipsychotics during the year preceding pregnancy, indicator of severity of psychiatric morbidity, indicator of tobacco use, indicator of alcohol use, folic acid, SSRI during pregnancy, child’s sex, gestational age and birth weight*

*** Cox models adjusted for: mother’s age, Complementary Universal Health Insurance scheme, indicator of tobacco use, indicator of alcohol use, folic acid, child’s sex, gestational age and birth weight*

Supplementary Table 15. Incidence of early neurodevelopmental disorders during follow-up according to exposure to topiramate during pregnancy, compared to unexposed children

|  | **Overall population** | | | | | |  | **Children born to a mother with no known mental illness** |
| --- | --- | --- | --- | --- | --- | --- | --- | --- |
|  | Children exposed to topiramate during pregnancy  (N=477) | |  | Unexposed children  (N=1,710,441) | | aHR [95%CI]* |  | aHR [95%CI]** |
|  | N | IR  per 1,000 PY |  | N | IR  per 1,000 PY |  |  |  |
| **Diagnosis** |  |  |  |  |  |  |  |  |
| Mental and behavioural disorders (F70-F98) | 7 | 4.1 |  | 15,270 | 2.5 | 1.2 [0.6 - 2.6] |  | 0.5 [0.1 - 3.7] |
| Pervasive developmental disorders (F84) | 1 | 0.6 |  | 4,280 | 0.7 | 0.7 [0.1 - 4.7] |  | - |
| Mental retardation (F70-F79) | 2 | 1.2 |  | 3,398 | 0.6 | 1.7 [0.4 - 6.8] |  | 2.2 [0.3 - 15.9] |
| Disorders of psychological development (F80-F89) | 3 | 1.7 |  | 10,010 | 1.6 | 0.8 [0.3 - 2.6] |  | - |
| Behavioural and emotional disorders with onset usually occurring in childhood and adolescence (F90-F98) | 4 | 2.3 |  | 4,398 | 0.7 | 2.1 [0.8 - 5.7] |  | - |
| **Health care utilization** |  |  |  |  |  |  |  |  |
| Speech therapy | 33 | 19.4 |  | 72,012 | 11.9 | **1.4 [1.0 - 2.0]^a^** |  | 1.5 [0.9 - 2.6] |
| Orthoptics | 70 | 44.5 |  | 203,489 | 35.6 | 1.2 [0.9 - 1.5] |  | 1.2 [0.8 - 1.7] |
| Psychiatry | 13 | 7.6 |  | 22,365 | 3.7 | 1.5 [0.9 - 2.5] |  | 1.2 [0.4 - 3.7] |

*IR: Incidence rate IRR: Incidence rate ratio HR: Hazard ratio 95%CI: 95% confidence interval*

** Cox models adjusted for: mother’s age, Complementary Universal Health Insurance scheme, diagnosis of mental illness other than tobacco and alcohol use disorders, antipsychotics during the year preceding pregnancy, indicator of severity of psychiatric morbidity, indicator of tobacco use, indicator of alcohol use, folic acid, SSRI during pregnancy, child’s sex, gestational age and birth weight*

*** Cox models adjusted for: mother’s age, Complementary Universal Health Insurance scheme, indicator of tobacco use, indicator of alcohol use, folic acid, child’s sex, gestational age and birth weight*

*^a^ p=0.03*
